# The Evolutionary History of Siphonophore Tentilla: Novelties, Convergence, and Integration

**DOI:** 10.1093/iob/obab019

**Published:** 2021-05-26

**Authors:** A Damian-Serrano, S H D Haddock, C W Dunn

**Affiliations:** 1 Osborn Memorial Laboratories, Department of Ecology and Evolutionary Biology, Yale University, New Haven, CT 06511, USA; 2 Midwater Research, Monterey Bay Aquarium Research Institute, Moss Landing, CA 95039, USA

## Abstract

**Synopsis** Siphonophores are free-living predatory colonial hydrozoan cnidarians found in every region of the ocean. Siphonophore tentilla (tentacle side branches) are unique biological structures for prey capture, composed of a complex arrangement of cnidocytes (stinging cells) bearing different types of nematocysts (stinging capsules) and auxiliary structures. Tentilla present an extensive morphological and functional diversity across species. While associations between tentillum form and diet have been reported, the evolutionary history giving rise to this morphological diversity is largely unexplored. Here we examine the evolutionary gains and losses of novel tentillum substructures and nematocyst types on the most recent siphonophore phylogeny. Tentilla have a precisely coordinated high-speed strike mechanism of synchronous unwinding and nematocyst discharge. Here we characterize the kinematic diversity of this prey capture reaction using high-speed video and find relationships with morphological characters. Since tentillum discharge occurs in synchrony across a broad morphological diversity, we evaluate how phenotypic integration is maintaining character correlations across evolutionary time. We found that the tentillum morphospace has low dimensionality, identified instances of heterochrony and morphological convergence, and generated hypotheses on the diets of understudied siphonophore species. Our findings indicate that siphonophore tentilla are phenotypically integrated structures with a complex evolutionary history leading to a phylogenetically-structured diversity of forms that are predictive of kinematic performance and feeding habits.

## Spanish

Los sifonóforos son cnidarios hidrozoos coloniales depredadores que habitan todas las regiones pelágicas del océano. Las téntilas (ramificaciones laterales de los tentáculos) de los sifonóforos son estructuras biológicas dedicadas a la captura de presas. Las téntilas se componen de una matriz compleja de cnidocitos (células urticantes) portadores de diferentes tipos de nematocistes (cápsulas urticantes) y estructuras auxiliares. Las téntilas presenta una extensa diversidad morfológica y funcional en las diferentes especies de sifonóforo. Las relaciones entre la forma de las téntilas y las dietas de los sifonóforos has sido estudiadas previamente, sin embargo, la historia evolutiva que dio lugar a esta diversidad morfológica no ha sido explorada apenas. En este estudio examinamos las adquisiciones y pérdidas evolutivas de las subestructuras de la téntila y los tipos de nematocisto utilizando la filogenia molecular más reciente de los sifonóforos. Las téntilas presentan un mecanismo de disparo a alta velocidad, sincronizando las diferentes subestructuras con gran precisión, durante el cual se la téntila de desenrolla mientras los nematocistes se disparan sobre la presa. En este estudio caracterizamos la diversidad cinemática de estas reacciones para la captura de presas utilizando video de alta velocidad, y describimos su relación con los caracteres morfológicos. Dado que la descarga de las téntilas ocurre en sincronía en toda su diversidad morfológica, hemos evaluado cómo la evolución con integración fenotípica mantiene las correlaciones entre los caracteres morfológicos a través del tiempo. Hallamos que el morfo-espacio de las téntilas tiene baja dimensionalidad, encontramos casos de heterocronía y convergencia morfológica, y generamos hipótesis sobre las dietas de especies de sifonóforo poco estudiadas. Nuestros hallazgos indican que las téntilas de los sifonóforos son estructuras con fenotipos integrados y con una historia evolutiva compleja que ha dado lugar a una diversidad filogenéticamente estructurada de formas asociadas a diferentes rendimientos cinemáticos y hábitos alimenticios.

## Introduction

**Fig. 1.**
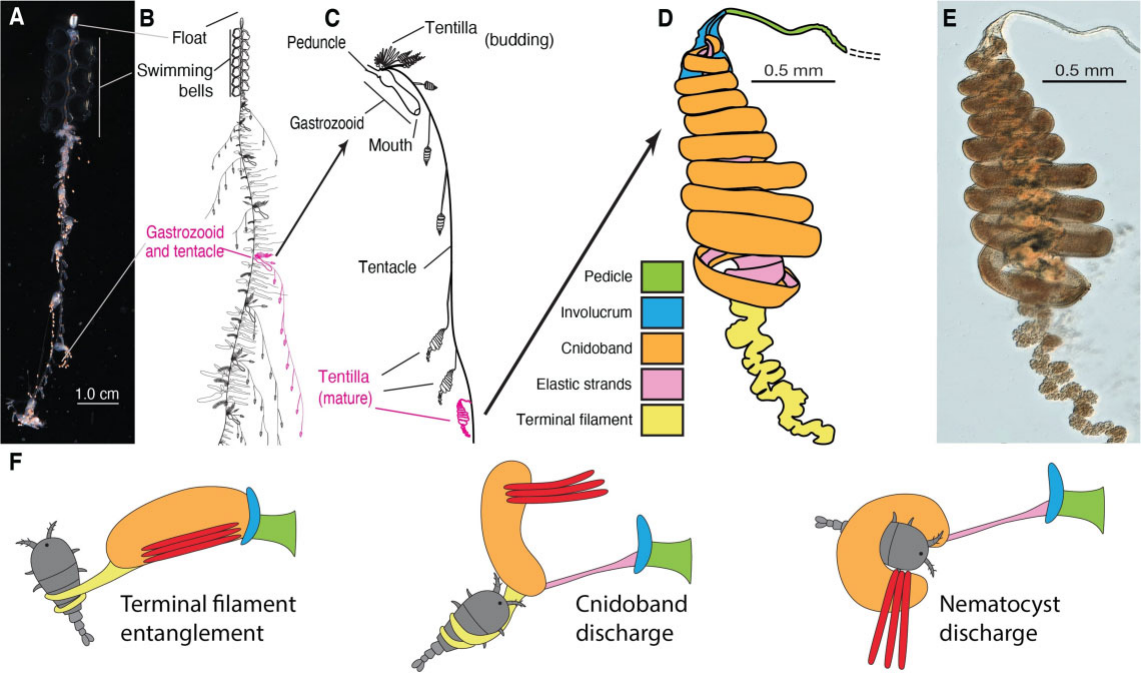
Siphonophore anatomy. (**A**) *Nanomia* sp. siphonophore colony (photo by Catriona Munro). (**B, C**) Illustration of a *Nanomia* colony, gastrozooid, and tentacle closeup (by Freya Goetz). (**D**) *Nanomia* sp. Tentillum illustration and main parts. (**E**) Differential interference contrast micrograph of the tentillum illustrated in (**D**) (Specimen: YPM IZ 106704). Figure reproduced from Damian-Serrano et al. (2021) with permission. (**F**) Action strip showing the behavior of tentilla during prey capture, illustrated by Riley Thompson.

Siphonophores have fascinated zoologists for centuries for their extremely subspecialized colonial organization and integration. Today we have a comprehensive taxonomic coverage on the morphological diversity of this group due to the extensive work of siphonophore taxonomists in the past few decades (Pugh 1983, 2001; Pugh and Harbison 1986; Pugh and Youngbluth 1988; Dunn et al. 2005; Haddock et al. 2005; Hissmann 2005; Bardi and Marques 2007; Pugh and Haddock 2010; Pugh and Baxter 2014), which has been elegantly synthesized in detailed synopses (Totton and Bargmann 1965; Mapstone 2014). In addition, recent advances in phylogenetic analyses of siphonophores (Munro et al. 2018; Damian-Serrano et al. 2021) have provided a macroevolutionary context to interpret this diversity. With these assets in hand, we can now begin to study siphonophores from a comparative perspective across taxa, focusing on the diversity and evolutionary history of specific structures. Here we focus on one such structure: the tentillum. Like many cnidarians, siphonophores bear tentacle side branches (tentilla) with nematocysts (Fig. 1C–E). But unlike other cnidarians, most siphonophore tentilla are dynamic structures that react to prey encounters by rapidly unfolding the nematocyst battery to slap around the prey (Fig. 1F). The acrorhagi in some anthozoans can be autonomously reactive (Williams 1991), but nowhere close to the complexity, speed, and coordination of tentillum discharge. This maximizes the surface area of contact between the nematocysts and the prey they fire upon.

**Fig. 2.**
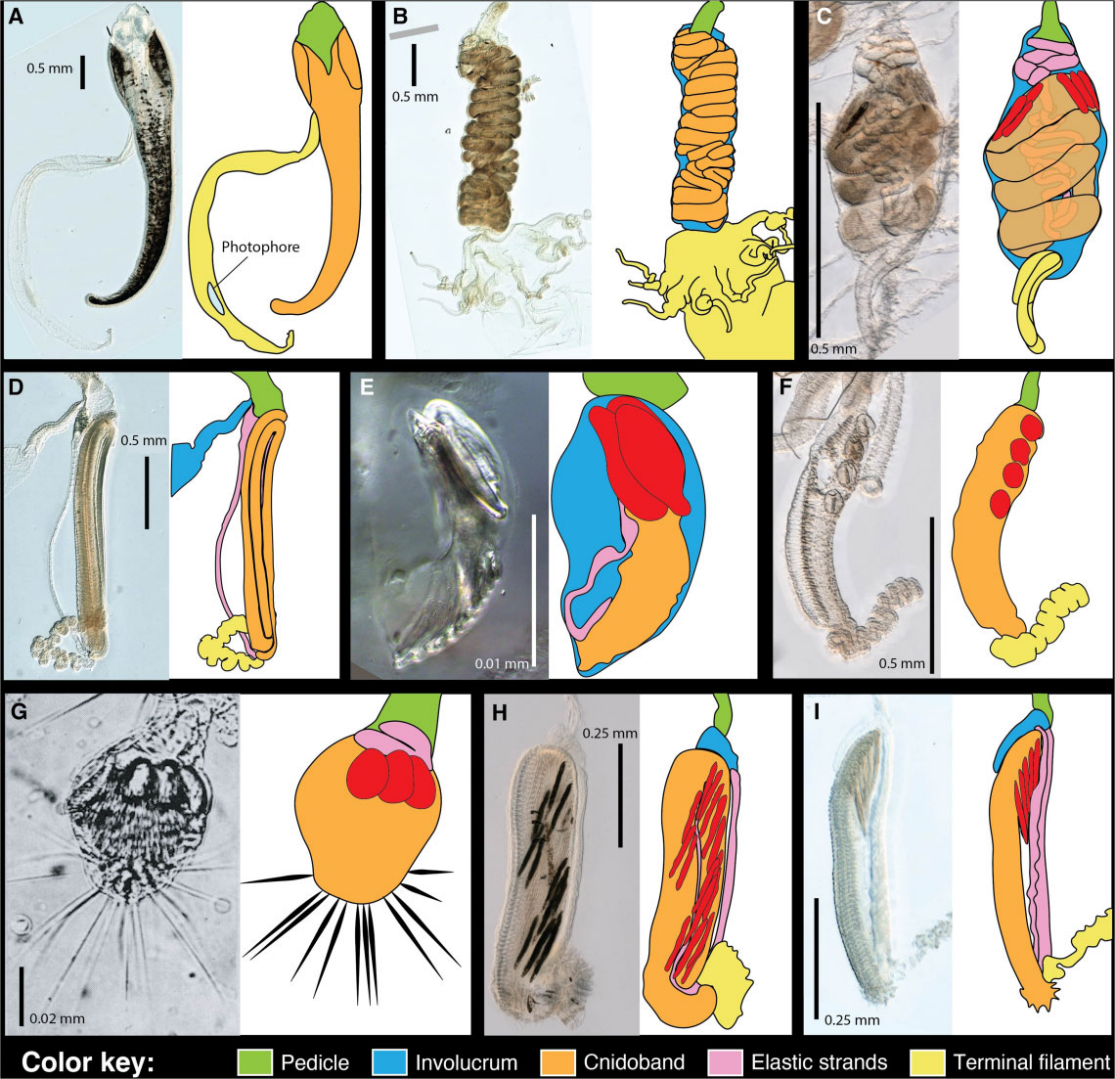
Tentillum diversity. The illustrations delineate the pedicle, involucrum, cnidoband, elastic strands, and terminal structures. Heteroneme nematocysts (stenoteles in **C**, **E**, **F**, and **G** and mastigophores in **H** and **I**) are only depicted for some species. (**A**) *Erenna laciniata* bears giant tentilla with a flicking bioluminescent lure, 10×. (**B**) *Lychnagalma utricularia* has a large convoluted cnidoband and unique buoyant medusa-shaped vesicle, 10×. (C) *Agalma elegans* has dual terminal filaments and ampulla, 10×. (**D**) *Resomia ornicephala* presents a zig–zag cnidoband and flap-shaped fluorescent involucrum, 10×. (E) *F. vityazi* has a minute encapsulated cnidoband with just three stenoteles, 20×. (F) *Bargmannia amoena* presents a simple tentillum with massive round stenoteles, 10×. (G) *Cordagalma* sp. has a greatly reduced tentillum with long terminal cnidocils (nematocyst-triggering sensory cilia), reproduced from Carré (1968). (H) *Lilyopsis fluoracantha* tentilla bear a pleated cnidoband flanked by long mastigophores, 20×. (I) *Abylopsis tetragona* exemplifies a typical calycophorans tentillum with desmonemes clustered at the distal end of the cnidoband, 20×.

**Fig. 3.**
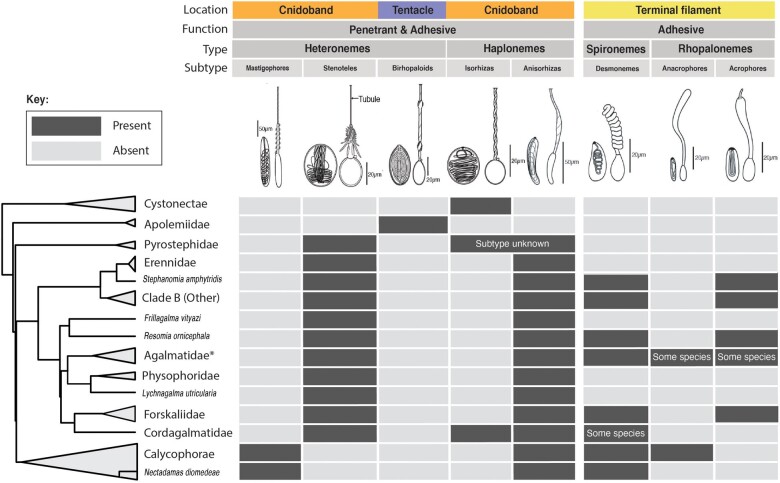
Phylogenetic distribution of nematocyst types, subtypes, functions, and locations in the tentacle across the major siphonophore clades. Illustrations reproduced with permission from Mapstone (2014). Undischarged capsules to the left, discharged to the right. Agalmatidae* here refers only to the genera *Agalma*, *Athorybia*, *Halistemma*, and *Nanomia*.

Siphonophore tentilla are defined as lateral, monostichous (branching on one side only) evaginations of the tentacle (including its gastrovascular lumen), armed with epidermal nematocysts (Totton and Bargmann 1965). The most complex ones are typically composed of (1) a flexible pedicle that provides the connection to the tentacle, (2) an epidermis-derived cnidoband that contains the penetrant and entangling haploneme and heteroneme nematocysts, (3) a rigid mesoglea-derived, collagen-based strand (called “elastic strand” though not very elastic) that runs ascending parallel and attached to the cnidoband with a descending portion detached from the cnidoband but firmly attached to the pedicle and the distal end of the cnidoband, (4) a terminal filament loaded with adhesive desmoneme and rhopaloneme nematocysts, and (5) an epithelial expansion named “involucrum” that arises from the pedicle and in some cases can completely cover the cnidoband (Figs. 1D and 2). A gastrodermis-derived axial tube is occasionally present in the cnidoband, but is often greatly reduced in the terminal filament (Totton and Bargmann 1965; Mackie et al. 1987; Mapstone 2014). The complexity of these structures varies greatly across siphonophores, yet the evolutionary history of this complexity remains unexplored. Tentillum discharge is typically elicited by adhesion of prey onto the terminal filament. During tentillum discharge, the distal end of the cnidoband shoots out, sometimes directed forward by the involucrum. The proximal end of the cnidoband detaches from the pedicle and slings forward. Nematocysts discharge as they come in contact with the surface of the prey, with the proximal heteronemes being the last ones to make contact. The structural integrity of the line connecting the tentacle to the prey for reeling is maintained by the elastic strand attachment to the cnidoband and pedicle (Fig. 1F). In addition, siphonophore tentilla present a remarkable diversity of morphologies (Fig. 2), sizes, and nematocyst complements (Fig. 3). In Fig. 2, we showcase a few of these different morphologies. Our overarching aim is to organize all this phenotypic diversity in a phylogenetic context, and identify the evolutionary processes that generated it.

Nematocysts are unique biological weapons for defense and prey capture exclusive to Cnidaria. Mariscal (1974) reported that hydrozoans have the largest diversity of nematocyst types among cnidarians. Among them, siphonophores present the greatest variety of types (Mapstone 2014), and vary widely across taxa in which and how many types they carry on their tentacles (Fig. 3). Werner (1965) noted that there are nine types of nematocyst found in siphonophores, of which four, anacrophore rhopalonemes, acrophore rhopalonemes, homotrichous anisorhizas, and birhopaloids, are unique to them. Heteroneme and haploneme nematocysts serve penetrant and entangling functions, while rhopalonemes and desmonemes work by adhering to the surface of the prey. While recent descriptive studies have expanded and confirmed our understanding of this diversity, the evolutionary history of nematocyst type gain and loss in siphonophores remains unexplored. Thus, here we reconstruct the evolution of shifts, gains, and losses of nematocyst types, subtypes, and other major categorical traits that led to the extant diversity we see in siphonophore tentilla.

Distantly related organisms that evolved to feed on similar resources often evolve similar adaptations (Winemiller et al. 2015). In Damian-Serrano et al. (2021), we found strong associations between piscivory and haploneme shape (elongation) across distantly related siphonophore lineages. These associations could have been produced by convergent changes in the adaptive optima of these characters. Here we set out to test this hypothesis using comparative model fitting methods. Analyzing the diversity of morphological states from a phylogenetic perspective allows us to identify the specific evolutionary processes that gave rise to it. Here we fit and compare a variety of macroevolutionary models to morphological measurement data from siphonophore tentilla to identify instances of neutral divergence, stabilizing selection, changes in the speed of evolution, and convergent evolution.

In Damian-Serrano et al. (2021), we fit discriminant analyses to identify characters that are predictive of feeding guild. These discriminant analyses can be used to generate hypotheses on the diets of ecologically understudied siphonophore species for which we have morphology data. Here we present a Bayesian prediction for the feeding guild of 45 species using the discriminant functions and morphological dataset in Damian-Serrano et al. (2021). As mentioned above, tentilla are far from being passive structures and are in fact violently reactive weapons for prey capture (Mackie et al. 1987; Damian-Serrano 2021; Damian-Serrano et al. 2021). While we now have detailed characterizations of tentillum morphologies across many species, the diversity of dynamic performances and their relationships to the undischarged morphologies have not been examined to date. To address this gap, we set out to record high-speed video of the *in vivo* discharge dynamics of several siphonophore species at sea (Damian-Serrano 2021), and compare the kinematic attributes to their morphological characters.

In Damian-Serrano et al. (2021), we collected a morphological dataset on siphonophore tentilla and nematocysts using microscopy techniques, and expanded the taxon sampling of the phylogeny to disentangle the evolutionary history. The analyses we carried out led to generalizable insights into the evolution of predatory specialization. The primary findings of that work were that generalists evolved from crustacean-specialist ancestors, and that feeding specializations were associated with distinct modes of evolution and character integration patterns. The work we present here is complementary to Damian-Serrano et al. (2021), showcasing a far more detailed account of the evolutionary history of tentillum morphology. In this study, we set out to examine seven core questions: (1) what is the evolutionary history of morphological novelties in siphonophore tentilla, (2) what models of evolution best describe the evolutionary history of tentillum and nematocyst characters, (3) are siphonophore tentilla phenotypically integrated, (4) does siphonophore feeding guild explain tentillum morphospace differentiation and disparity, (5) are any of the similarities between the tentilla of siphonophores in the same feeding guild convergent, (6) what prey should we expect understudied siphonophore species to feed upon based on their tentillum morphology, and (7) are there any differences in tentillum discharge performance predicted from tentillum morphology.

## Materials and methods

All character data and the phylogeny analyzed here were published in Damian-Serrano et al. (2021) and are available in the associated Dryad repository (Damian-Serrano et al. 2020). Details on the specimen collection, microscopy, and measurements can be found in the aforementioned publication. To facilitate access, we re-included here the character definitions (SM15) and specimen list (SM16) in the Supporting Information. We also made all the microscopy images available through the Yale Peabody Museum collections website (https://collections.peabody.yale.edu/). These images are flat projections of the z-stacks, which will be available upon request from the Invertebrate Zoology collection. In this dataset, multiple specimens of each species were measured when possible. For each specimen there was a single measurement taken of each character, giving a greater focus to capturing species and intraspecific specimen diversity than to capturing intra-individual variation. These measurements should not be used for diagnostic nor taxonomic purposes, since they do not capture the full span of intra-individual nor intra-specific variation. Since the goal of these morphological measurements was comparative and not diagnostic, it is not as relevant whether a specimen is representative of the taxon. Moreover, desmoneme, rhopaloneme, and heteroneme sizes are extremely uniform in siphonophore tentilla. For example, in the description of *Sphaeronectes haddocki* (Pugh 2009), they describe the mastigophore size range is 65.4 × 10.4 to 63.6 × 9.1 µm; or in Purcell (1984), *Agalma okenii* stenoteles are shown to range between 112.5 × 20 and 135 × 24 µm. The error margins on our mean values match the ranges measured in other published studies where multiple nematocysts were measured per specimen. Our evolutionary models and phylogenetic signal calculations incorporate these margins as standard errors. When a homologous nematocyst type had subspecialized into two forms or size classes (such as the isorhizas of cystonects, or the central *v.s.* edge cnidoband anisorhizas), only one class was consistently measured. We took the largest in the case of cystonect isorhizas, and the central ones in the case of cnidoband anisorhizas, since either class is homologous to the single class in other taxa. Due to the small intra-specific sample sizes, the normality of the measurement distributions within species could not be ascertained. We log-transformed all the continuous characters that did not pass Shapiro–Wilk’s normality tests across species, and used the ultrametric constrained Bayesian time tree in all comparative analyses. In the species measured for comparative analyses, between 3 and 11 specimens were typically measured (SM17) with the exception of *Agalma clausi*, *Chuniphyes moserae*, *Forskalia formosa*, *Forskalia tholoides*, *Kephyes ovata*, Physonect sp., and *Physophora gilmeri* with one specimen each, and *Erenna sirena* with two specimens. The number of specimens included per species was limited by specimen availability, since finding and collecting certain siphonophore species can be extremely challenging.

Inapplicable characters were recorded as NA states, and species with states that could not be measured due to technical limitations were removed before the analyses. We used the feeding guild categories detailed in Damian-Serrano et al. (2021) with one modification: including all *Forskalia* spp. as generalists instead of as a single *Forskalia* species on the tree after a reinterpretation of the data in Purcell (1981). In order to characterize the evolutionary history of tentillum morphology, we fitted different models generating the observed data distribution given the phylogeny for each continuous character using the function fitContinuous in the R package *geiger* (Harmon et al. 2008). These models include a non-phylogenetic white-noise model, a neutral divergence Brownian Motion (BM) model, an early-burst (EB) decreasing rate model, and an Ornstein–Uhlenbeck (OU) model with stabilizing selection around a fitted optimum trait value. In the same way as Damian-Serrano et al. (2021), we then ordered the models by increasing parametric complexity, and compared their corrected Akaike Information Criterion scores (Sugiura 1978). We used the lowest (best) score with a delta of 2 to determine significance relative to the next simplest model (SM10). We calculated model adequacy scores using the R package *arbutus* (Pennell et al. 2015) (SM11), and calculated phylogenetic signals in each of the measured characters using Blomberg’s K (Blomberg et al. 2003) (SM10). To reconstruct the ancestral character states of nematocyst types and other categorical traits (character matrix available in Supplementary Information), we used stochastic character mapping (SIMMAP) using the package *phytools* (Revell 2012).

In order to examine the degree of phenotypic integration within the tentillum, we explored the correlational structure among continuous characters and among their evolutionary histories using principal component analysis (PCA) and phylogenetic PCA (Revell 2012). Since the character dataset contains gaps due to missing data and inapplicable character states (SM14), we carried out these analyses on a subset of species and characters that allowed for the most complete dataset. This was done by removing the terminal filament characters (which are only shared by a small subset of species), and then removing species that had inapplicable states for the remaining characters (apolemiids and cystonects). In addition, we obtained the correlations between the phylogenetic independent contrasts (Felsenstein 1985) using the package *rphylip* (Revell and Chamberlain 2014) accounting for intraspecific variation. Using these contrasts, we identified multivariate correlational modules among characters. To test and quantify phenotypic integration between these multivariate modules, we used the phylogenetic phenotypic integration test in the package *geomorph* (Adams et al. 2018).

When comparing the morphospaces of species in different feeding guilds, we carried out a PCA on the complete character dataset while transforming inapplicable states of absent characters to zeros (i.e., cnidoband length = 0 when no cnidoband is present) to account for similarity based on character presence/absence. Using these principal components, we examined the occupation of the morphospace across species in different feeding guilds using a phylogenetic MANOVA (multivariate analysis of variance) with the package *geiger* (Harmon et al. 2008) to assess the variation explained, and a morphological disparity test with the package *geomorph* (Adams et al. 2018) to assess differences in the extent occupied by each guild.

In order to detect and evaluate instances of convergent evolution, we used the package SURFACE (Ingram and Mahler 2013). This tool identifies OU regimes and their optima given a tree and character data, and then evaluates where the same regime has appeared independently in different lineages. We applied these analyses to the haploneme nematocyst length and width characters as well as to the most complete dataset without inapplicable character states with 43 species and 186 specimens (SM17).

In order to generate hypotheses on the diets of siphonophores using tentillum morphology, we used the discriminant analyses of principal components (DAPCs; Jombart et al. 2010) trained in Damian-Serrano et al. (2021). We predict the feeding guilds of species in the dataset for which there are no published feeding observations using their morphological data as inputs, and presenting the predictive output in the form of posterior probabilities for each guild category.

To observe the discharge behavior of different tentilla, we recorded high speed footage (1000–3000 fps) of tentillum and nematocyst discharge by live siphonophore specimens (26 species) using a Phantom Miro 320S camera mounted on a stereoscopic microscope. We mechanically elicited tentillum and nematocyst discharge using a fine metallic pin. We used the Phantom PCC software to analyze the footage. For the 10 species recorded, we measured total cnidoband discharge time (ms), heteroneme filament length (μm), and discharge speeds (mm/s) for cnidoband, heteronemes, haplonemes, and heteroneme shafts when possible (all data and code are available in the Github repository https://github.com/dunnlab/tentilla_organismal/).

## Results

### Evolutionary history of tentillum morphology

The phylogeny of Damian-Serrano et al. (2021) had revealed for the first time that the genus *Erenna* is the sister to *Stephanomia amphytridis*. *Erenna* and *Stephanomia* bear the largest tentilla among all siphonophores, thus their monophyly indicates that there was a single evolutionary transition to giant tentilla. Siphonophore tentilla range in size from ∼30 µm in some *Cordagalma* specimens to 2–4 cm in *Erenna* species, and up to 8 cm in *S. amphytridis* (Pugh and Baxter 2014). Most siphonophore tentilla measure between 175 and 1007 µm (first and third quartiles), with a median of 373 µm. The extreme gain of tentillum size in this newly recognized clade may have important implications for access to large prey size classes such as adult deep-sea fishes.

**Fig. 4.**
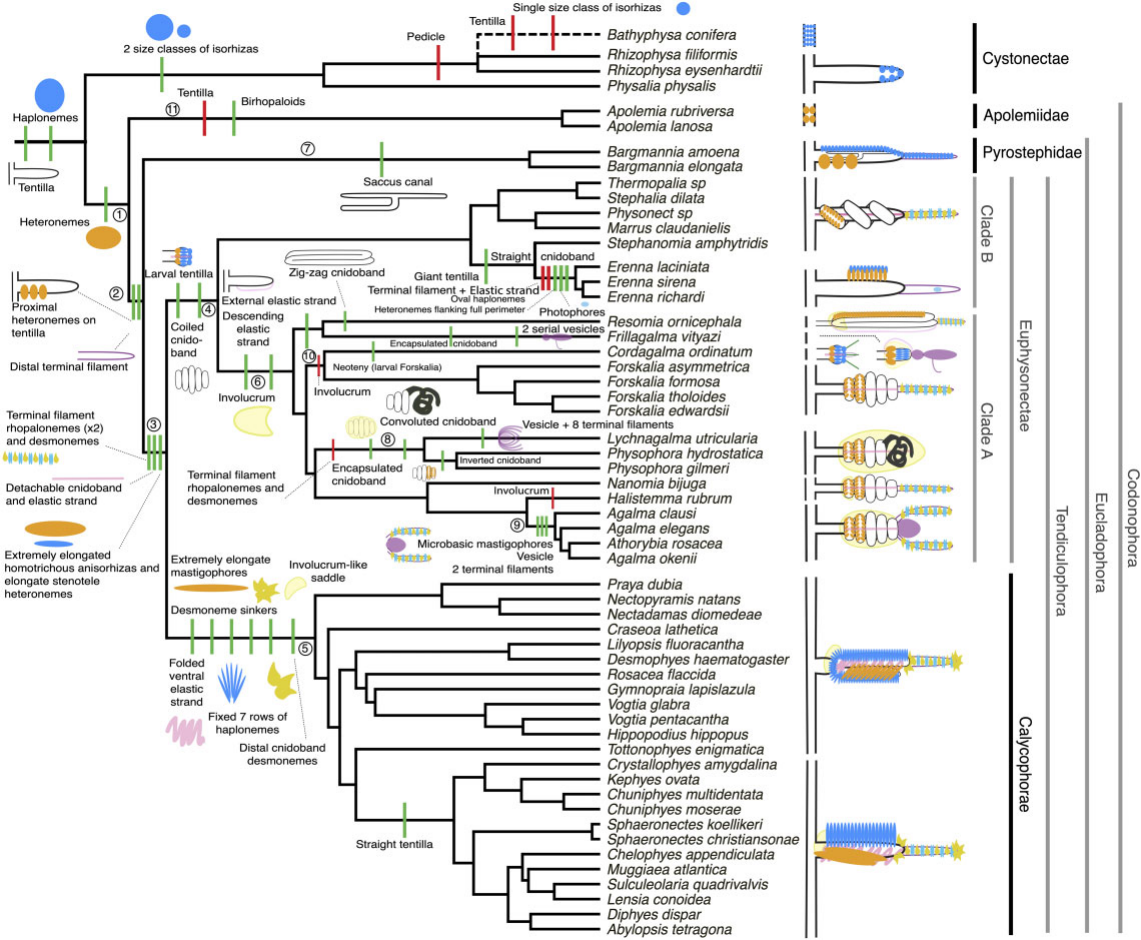
Siphonophore cladogram with the main categorical character gains (green) and losses (red) mapped. Some branch lengths were modified from the Bayesian chronogram to improve readability. The main visually distinguishable tentillum types are sketched next to the species that bear them, showing the location and arrangement of the main characters. In large, complex-shaped euphysonect tentilla, haplonemes were omitted for simplification. The hypothesized phylogenetic placement of the rhizophysid *Bathyphysa conifera*, for which no molecular data are yet available, was added manually (dashed line). Some branches have been numbered 1–11 to facilitate their reference in the text.

The buttons on *Physalia* tentacles (see one of our imaged specimens https://collections.peabody.yale.edu/search/Record/YPM-IZ-106663) were not traditionally regarded as tentilla, but Bardi and Marques (2007), Munro et al. (2018), and our own observations confirm that the buttons contain evaginations of the gastrovascular lumen, thus satisfying all the criteria for the definition given in the “Introduction” section. In this light, and given that most Cystonectae bear conspicuous tentilla, we conclude, in agreement with Munro et al. (2018), that tentilla were present in the most recent common ancestor of all siphonophores, and secondarily lost twice, once in *Apolemia* and again in *Bathyphysa conifera*. In order to gain a broad perspective on the evolutionary history of tentilla, we reconstructed the phylogenetic positions of the main categorical character shifts (such as gains and losses of nematocyst types) using stochastic character mapping (SM1-9) and manual reconstructions. This phylogenetic roadmap of evolutionary novelties is summarized in Fig. 4.

The phylogenetic position of siphonophores within Hydroidolina has been inconsistent across different studies. In Cartwright et al. (2008), they are reported as sister to Aplanulata, in Cartwright and Nawrocki (2010) they appear to be sister to Leptothecata, while in Kayal et al. (2015) they appear as sister to all other Hydroidolina. However, in the first two cases, the node support for these relationships is weak, and in the last case, the results are based on mitochondrial genes only. In Bentlage and Collins (2020), siphonophores appear as sister to the clade composed of Filifera III and Filifera IV, with strong node support. In any case, their affinities are congruent with the assumption that haploneme nematocysts are ancestrally present in siphonophore tentacles since they are present in the tentacles of many other hydrozoans (Mariscal 1974). Haplonemes are toxin-bearing open-ended nematocysts characterized by the lack of a shaft preceding the tubule. Two subtypes are found in siphonophores: the isorhizas of homogeneous tubule width, and the anisorhizas with a slight enlargement of the tubule near the base. In Cystonectae, haplonemes diverged into spherical isorhizas of two size classes. There is one size of haplonemes in Codonophora, which consist of elongated anisorhizas. Haplonemes were likely lost in the tentacles of *Apolemia* but retained as spherical isorhizas in other *Apolemia* tissues (Siebert et al. 2013). While heteronemes exist in other tissues of cystonects, they appear in the tentacles of codonophorans exclusively—as birhopaloids in *Apolemia*, stenoteles in eucladophoran physonects (except *Agalma* and *Athorybia* spp.), and microbasic mastigophores in calycophorans and in the *Agalma–Athorybia* clade. The four nematocyst types unique to siphonophores appear in two events in the phylogeny (Fig. 4): birhopaloids arose in the lineage leading to *Apolemia* (Fig. 4, branch 11), while rhopalonemes (acrophore and anacrophore) and elongated homotrichous anisorhizas arose in the lineage leading to Tendiculophora (Fig. 4, branch 3).

Nematocyst type gain and loss is also associated with prey capture functions. For example, the loss of desmonemes and rhopalonemes in piscivorous *Erenna*, retaining solely the penetrant (and venom injecting) anisorhizas and stenoteles (two size classes) is reminiscent of the two size classes of penetrant isorhizas in the fish-specialist cystonects. Moreover, with the gain of anisorhizas, desmonemes, and rhopalonemes, the Tendiculophora gained versatility in entangling and adhesive functions of the cnidoband and terminal filament, which may have allowed their feeding niches to diversify. Part of the effectiveness of calycophoran cnidobands at entangling crustaceans may be attributed to the subspecialization of their heteronemes. These shifted from the ancestral stenotele to the microbasic mastigophore (or eurytele in some species) with a long, barbed shaft armed with many long spines. This heteroneme subtype could be better at interlocking with and adhering to the setae of crustacean legs and antennae. In those species that have a functional terminal filament, the desmonemes and rhopalonemes play a fundamental role in the first stages of adhesion of the prey. In many species, the tugs of the struggling prey on the terminal filament trigger the cnidoband discharge (Mackie et al. 1987 and personal observation). The adhesive terminal filament has been lost several times in the Euphysonectae (*Frillagalma*, *Lychnagalma*, *Physophora*, *Erenna*, and some species of *Cordagalma*). In these species, we hypothesize that a different trigger mechanism is at play, possibly involving the prey actively biting or grasping the tentillum or lure.

The clades defined in Damian-Serrano et al. (2021) are characterized by unique evolutionary innovations in their tentilla. The clade Eucladophora (containing Pyrostephidae, Euphysonectae, and Calycophorae) encompasses all of the extant siphonophore species (178 of 186) except Cystonects and *Apolemia*. Innovations that arose along the lineage leading to this group (Fig. 4, branch 2) include spatially segregated heteroneme and haploneme nematocysts, terminal filaments, and elastic strands. Pyrostephids (Fig. 4, branch 7) evolved a unique bifurcation of the axial gastrovascular canal of the tentillum known as the “saccus” (Totton and Bargmann 1965). The lineage leading to the clade Tendiculophora (clade containing Euphysonectae and Calycophorae, see Fig. 4, branch 3) subsequently acquired further novelties such as the desmonemes and rhopalonemes (acrophore subtype present in euphysonects and anacrophore subtype present in calycophorans) on the terminal filament, which bears no other nematocyst type. These are arranged in sets of two parallel rhopalonemes for each single desmoneme (Skaer 1988, 1991). The involucrum is an expansion of the epidermal layer that can cover part or all of the cnidoband (Fig. 2). This structure, together with differentiated larval tentilla, appeared in the branch leading to Clade A physonects (Fig. 4, branch 6).

Among Clade A euphysonects, several interesting novelties have arisen. The clade composed of *Forskalia* and *Cordagalma* (Fig. 4, branch 10) lost their involucrum, while *Halistemma rubrum* had it greatly reduced to a vestigial form. Other *Halistemma* species have retained their ancestral involucrum (Mapstone 2003; Pugh and Baxter 2014). *Frillagalma* lost its terminal filament, and gained an encapsulated cnidoband (cnidosac) followed by their characteristic serial, fluid-filled, vesicles which may act as a lure for prey. The branch leading to the clade comprising *Lychnagalma* and *Physophora* (Fig. 4, branch 8) similarly encapsulated their cnidoband—losing their terminal filament and shifting the coiled cnidoband shape to a much more convoluted morphology. *Lychnagalma* subsequently gained its characteristic floating medusa-shaped vesicle, while *Physophora* completely inverted the orientation of its cnidoband, placing its heteronemes near the distal end. The clade composed of *Agalma* and *Athorybia* (Fig. 4, branch 9) modified their terminal filament into two thick terminal filaments with minute rhopaloneme nematocysts separated by a central, fluid-filled ampulla.

Calycophorans evolved novelties such as larger desmonemes at the distal end of the cnidoband, pleated pedicles with a “hood” (here considered homologous to the involucrum) at the proximal end of the tentillum, anacrophore rhopalonemes, and microbasic mastigophore-type heteronemes (Fig. 4, branch 5). While calycophorans have diversified into most of the extant described siphonophore species (108 of 186), their tentilla have not undergone any major categorical gains or losses since their most recent common ancestor. Nonetheless, they have evolved a wide variation in nematocyst and cnidoband sizes. Ancestrally (and retained in most prayomorphs and hippopodiids), the calycophoran tentillum is recurved where the proximal and distal ends of the cnidoband are close together. Diphyomorph tentilla are slightly different in shape, with straighter cnidobands.

### Evolution of tentillum and nematocyst characters

Most (74%) characters present a significant phylogenetic signal, yet only total nematocyst volume, haploneme length, and heteroneme-to-cnidoband length ratio had a phylogenetic signal with *K* > 1 (SM10). Total nematocyst volume and cnidoband-to-heteroneme length ratio showed strongly conserved phylogenetic signals. The majority (67%) of log-transformed characters were best fitted by BM models, indicating a history of neutral constant divergence. We did not find any relationship between phylogenetic signal and specific model support, where characters with high and low phylogenetic signal were broadly distributed among the best fitted for each model. One-third of the characters measured in Damian-Serrano et al. (2021) did not recover significant support for any of the phylogenetic models tested, indicating they are either not phylogenetically conserved, or they evolved under a complex evolutionary process not represented among the models tested (SM10). Haploneme nematocyst length was the only character with support for an EB model of decreasing rate of evolution with time. No character had support for a single-optimum OU model (when not informed by feeding guild regime priors). The model adequacy tests (SM11) indicate that many characters may have a relationship between the states and the rates of evolution (Sasr) not captured in the basic models compared here, accompanied by a signal of unaccounted rate heterogeneity (Cvar). No characters show significant deviations in the overall rate of evolution estimated (Msig). Some characters show a perfect fit (no significant deviations across all metrics) under BM evolution, such as heteroneme elongation, length, width and volume, haploneme width and SA/V, tentacle width, and pedicle width. Haploneme row number and rhopaloneme elongation have significant deviations across four metrics, indicating that best model (BM) is a poor fit. These characters likely evolved under complex models which would require many more data points than we have available to fit with accuracy.

### Phenotypic integration of the tentillum

**Fig. 5.**
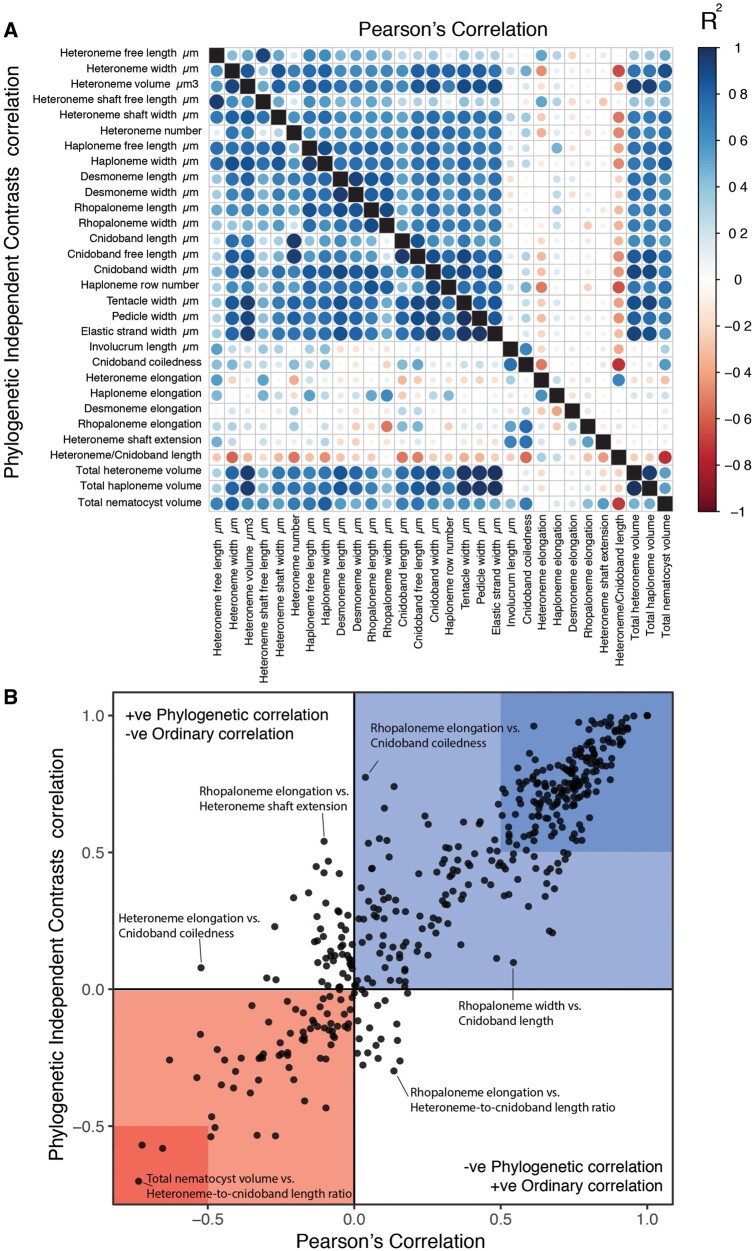
(**A**) Correlogram showing strength of ordinary (upper triangle) and phylogenetic (lower triangle) correlations between characters. Both size and color of the circles indicate the strength of the correlation (*R*^2^). (**B**) Scatterplot of phylogenetic correlation against ordinary correlation showing a strong linear relationship (*R*^2^ = 0.92, 95% confidence between 0.90 and 0.93). Light red and blue boxes indicate congruent negative and positive correlations, respectively. Darker red and blue boxes indicate strong (<−0.5 or >0.5) negative and positive correlation coefficients, respectively.

Phenotypically integrated structures maintain evolutionary correlations between their constituent characters. Of the phylogenetic correlations among tentillum and nematocyst characters examined here (Fig. 5A, lower triangle), 81.3% were positive and 18.7% were negative, while of the ordinary correlations (Fig. 5A, upper triangle) 74.6% were positive and 25.4% were negative. Half (49.9%) of phylogenetic correlations were >0.5, while only 3.6% are <−0.5. Similarly, among the correlations across extant species, 49.1% were >0.5 and only 1.5% were <−0.5. In addition, we found that 13.9% of character pairs had opposing phylogenetic and ordinary correlation coefficients (Fig. 5B). Just 4% of character pairs have negative phylogenetic and positive ordinary correlations (such as rhopaloneme elongation ∼ heteroneme-to-cnidoband length ratio and haploneme elongation, or haploneme elongation ∼ heteroneme number), and only 9.9% of character pairs had positive phylogenetic correlation yet negative ordinary correlation (such as heteroneme elongation ∼ cnidoband convolution and involucrum length, or rhopaloneme elongation with cnidoband length). These disparities could be explained by Simpson’s paradox (Blyth 1972): the reversal of the sign of a relationship when a third variable (or a phylogenetic topology, as suggested by Uyeda et al. [2018]) is considered. However, no character pair had correlation coefficient differences >0.64 between ordinary and phylogenetic correlations (heteroneme shaft extension ∼ rhopaloneme elongation has a Pearson’s correlation of 0.10 and a phylogenetic correlation of −0.54). Rhopaloneme elongation shows the most incongruence between phylogenetic and ordinary correlations with other characters. We identified four hypothetical modules among the tentillum characters: (1) The tentillum scaffold module including cnidoband length and width, nematocyst row number, pedicle and elastic strand width, tentacle width; (2) the heteroneme module including heteroneme length and width, shafts length and width; (3) the haploneme module including length and width; and (4) the terminal filament module including desmoneme and rhopaloneme length and width. The phenotypic integration test showed significant integration signal between all modules, tentillum, and haploneme modules sharing the greatest regression coefficient (SM12).

### Evolution of nematocyst shape

**Fig. 6.**
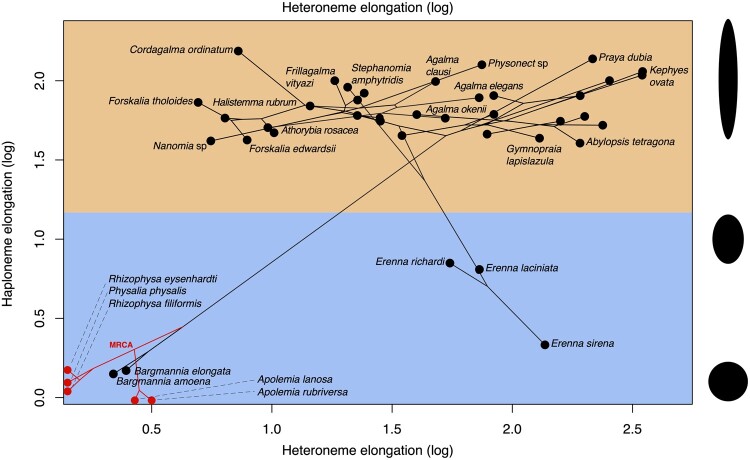
Phylomorphospace showing haploneme and heteroneme elongation (log scaled). Orange area delimits rod-shaped haplonemes, the blue area covers oval and round-shaped haplonemes. Smaller dots and lines represent phylogenetic relationships and ancestral states of internal nodes under BM. Species nodes in red lack either haplonemes or heteronemes, and their values are projected onto the axis of the nematocyst type they bear. Cystonects have no tentacle heteronemes and are projected onto the haploneme axis. Apolemiids have no tentacle haplonemes and are projected onto the heteroneme axis. Silhouettes on the right side represent haploneme shapes along the *y*-axis.

The greatest evolutionary change in haploneme nematocyst shape occurred in a single shift toward elongation in the branch leading to Tendiculophora, which contains the majority of described siphonophore species, that is, all siphonophores other than Cystonects, *Apolemia*, and Pyrostephidae. There is one secondary return to more oval, less elongated haplonemes in *Erenna*, but it does not reach the sphericity present in Cystonectae or Pyrostephidae (Fig. 6). Heteroneme evolution presents a less discrete evolutionary history. Tendiculophora evolved more elongate heteronemes before diversifying, but the difference between theirs and other siphonophores is much smaller than the variation in elongation within Tendiculophora, bearing no phylogenetic signal within this clade. In this clade, the evolution of heteroneme elongation has diverged in both directions, and there is no correlation with haploneme elongation (Fig. 6), which has remained fairly constant (elongation between 1.5 and 2.5).

**Fig. 7.**
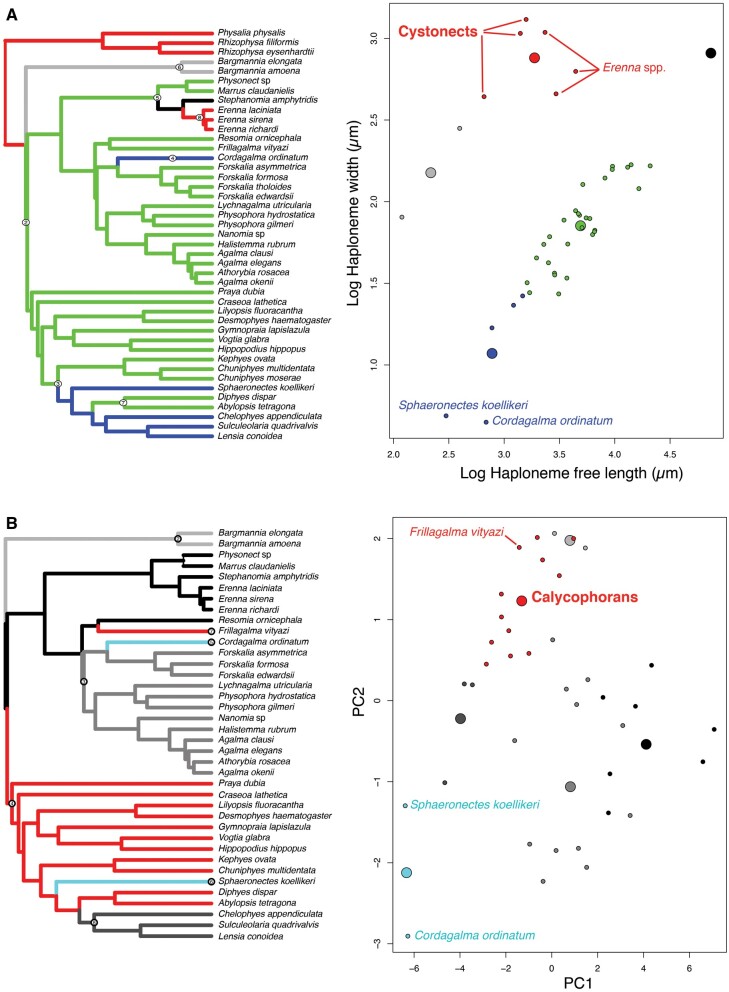
SURFACE plots showing convergent evolutionary regimes modelled under OU for (**A**) haploneme nematocyst length and width, and (**B**) for PC1 and PC2 of all continuous characters with the exception of terminal filament nematocysts, and removing taxa with inapplicable character states. Node numbers on the tree label different regimes, regimes of the same color are identified as convergent. Small circles on the scatterplots indicate species values, large circles indicate the average position of the OU optima (*theta*) for a given combination of convergent regimes.

Haploneme and heteroneme elongation share 21% of their variance across extant values, and 53% of the variance in their shifts along the branches of the phylogeny. However, much of this correlation is due to the sharp contrast between Pyrostephidae and their sister group Tendiculophora. We searched for regime shifts in the evolution of haploneme nematocyst length and width using SURFACE (Ingram and Mahler 2013). SURFACE identified eight distinct OU regimes in the evolutionary history of haploneme length and width (Fig. 7A). The different regimes are located in (1) cystonects, (2) most of Tendiculophora, (3) most diphyomorphs, (4) *Cordagalma ordinatum*, (5) *S. amphytridis*, (6) pyrostephids, (7) *Diphyes dispar* + *Abylopsis tetragona*, and (8) *Erenna* spp.

**Fig. 8.**
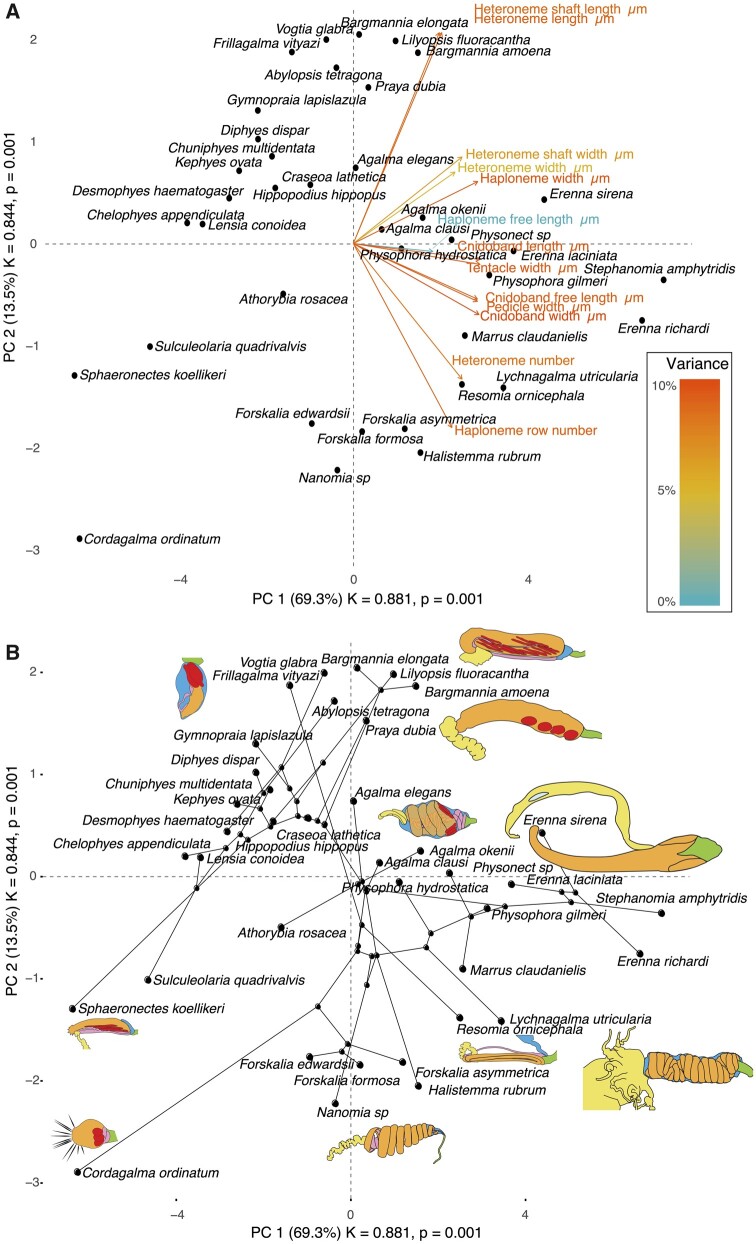
PCA of the simple-measurement continuous characters principal components, excluding ratios and composite characters. (**A**) Variance explained by each variable in the PC1–PC2 plane. Axis labels include the phylogenetic signal (K) for each component and *P*-value. (**B**) Phylogenetic relationships between the species points and reconstructed ancestors distributed in that same space.

In the non-phylogenetic PCA morphospace using only characters derived from simple measurements (Fig. 8), PC1 (aligned with tentillum and tentacle size) explained 69.3% of the variation in the tentillum morphospace, whereas PC2 (aligned with heteroneme length, heteroneme number, and haploneme arrangement) explained 13.5%. In a phylogenetic PCA, 63% of the evolutionary variation in the morphospace is explained by PC1 (aligned with shifts in tentillum size), while 18% is explained by PC2 (aligned with shifts in heteroneme number and involucrum length).

### Morphospace occupation

**Fig. 9.**
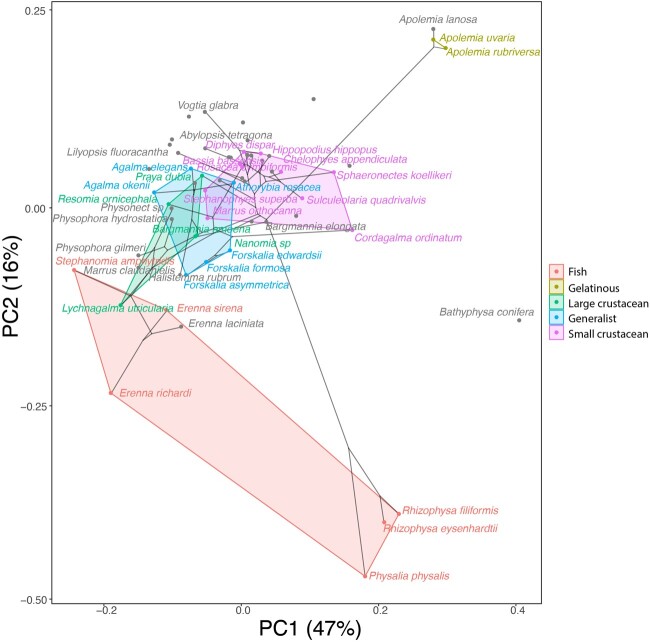
Phylomorphospace showing PC1 and PC2 from a PCA of continuous morphological characters with inapplicable states transformed to zeroes, overlapped with polygons conservatively defining the space occupied by each feeding guild. Lines between species coordinates show the phylogenetic relationships between them. Gray points indicate species with no feeding guild information.

In order to examine the occupation structure of the morphospace across all siphonophore species in the dataset, we cast a PCA on the data after transforming inapplicable states (due to absence of character) to zeroes. This allows us to accommodate species with many missing characters (such as cystonects or apolemiids), and to account for common absences as morphological similarities. In this ordination, PC1 (aligned with cnidoband size) explains 47.45% of variation and PC2 (aligned with heteroneme volume and involucrum length) explains 16.73% of variation. When superimposing feeding guilds onto the morphospace (Fig. 9), we find that the morphospaces of each feeding guild are only slightly overlapping in PC1 and PC2. A phylogenetic MANOVA showed that feeding guilds explain 27.63% of variance across extant species (*P*-value < 0.000001), and 20.97% of the variance when accounting for phylogeny, an outcome significantly distinct from the expectation under neutral evolution (*P*-value = 0.0196). In addition, a morphological disparity analysis accounting for phylogenetic structure shows that the morphospace of fish specialists is significantly broader than that of generalists and other specialists, and the gelatinous morphospace is significantly smaller than that of all other feeding guilds. This is mainly due to the large morphological disparities between cystonects and piscivorous euphysonects, and to the narrow taxonomic diversity of gelatinous specialists (*Apolemia* spp.). There are no significant differences among the morphospace disparities of the other feeding guilds.

### Convergent evolution

Convergence is a widespread evolutionary phenomenon where distantly related clades independently evolve similar phenotypes. When the dimensionality of the state space is small as it is in tentilla morphology, convergence is more likely given the same amount of evolutionary change. Using the package SURFACE (Ingram and Mahler 2013), we identified convergence in haploneme nematocyst dimensions and in morphospace positions. In Damian-Serrano et al. (2021), we identified haploneme nematocyst shape as one of the traits associated with the convergent evolution of piscivory. Here we find that indeed wider haploneme nematocysts have convergently evolved in the piscivorous cystonects and *Erenna* spp. (Fig. 7A). Independent shifts in width are responsible for this convergent loss of elongation. When integrating many traits into a couple principal components, we find two distinct convergences between euphysonects and calycophorans with a reduced prey capture apparatus. Those convergences are between *Frillagalma vityazi* and calycophorans, and between the extremely small haplonemes in the euphysonect *C. ordinatum* and copepod specialist calycophorans such as *Sphaeronectes koellikeri* (Fig. 7B).

### Functional morphology of tentillum and nematocyst discharge

Tentillum and nematocyst discharge high speed videos and measurements are available in the Supplementary Data. While the sample sizes of these measurements were insufficient to draw reliable statistical results at a phylogenetic level, we did observe patterns that may be relevant to their functional morphology. For example, cnidoband length is strongly correlated with discharge speed (*P*-value = 0.0002). This explains much of the considerable difference between euphysonect and calycophoran tentilla discharge speeds (average discharge speeds: 225.0 and 41.8 mm/s, respectively; *t*-test *P*-value = 0.011), since the euphysonects have larger tentilla than the calycophorans among the species recorded. In addition, we observed that calycophoran haploneme tubules fire faster than those of euphysonects (*t*-test *P*-value = 0.001). Haploneme nematocysts discharge 2.8× faster than heteroneme nematocysts (*t*-test *P*-value = 0.0012). Finally, while all nematocysts evert a twisted filament in a subtle solenoid motion, we observed that the stenotele filament of the Euphysonectae discharges in a distinctively coiled solenoid fashion that “drills” itself like a corkscrew through the medium it penetrates as it everts. This is particularly conspicuous in the stenoteles of *F. vityazi* (Damian-Serrano 2021), and is very different from how typical nematocysts, such as *Hydra* stenoteles, evert (Holstein and Tardent 1984; Nüchter et al. 2006).

### Generating dietary hypotheses using tentillum morphology

**Fig. 10.**
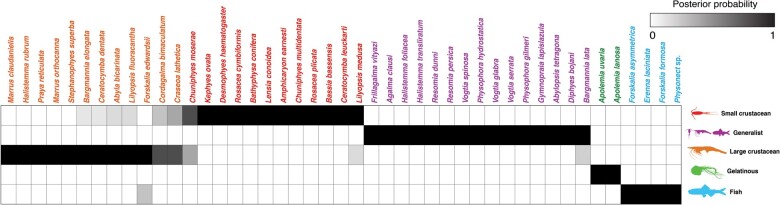
Hypothetical feeding guilds for siphonophore species predicted by six PCA DAPC. Cell darkness indicates the posterior probability of belonging to each guild. The training dataset was transformed so inapplicable states are computed as zeroes. Species are sorted and colored according to their predicted feeding guild.

For many siphonophore species, no feeding observations have yet been published. To help bridge this gap of knowledge, we generated hypotheses about the diets of these understudied siphonophores (Fig. 10) based on their known tentacle morphology using one of the linear DAPCs fitted in Damian-Serrano et al. (2021). This provides concrete predictions to be tested in future work and helps extrapolate our findings to many poorly known species that are extremely difficult to collect and observe. The discriminant analysis for feeding guild (seven principal components, four discriminants) produced 100% discrimination, and the highest loading contributions were found for the characters (ordered from highest to lowest): Involucrum length, heteroneme volume, heteroneme number, total heteroneme volume, tentacle width, heteroneme length, total nematocyst volume, and heteroneme width. We used the predictions from this discriminant function to generate hypotheses about the feeding guild of 45 species in the morphological dataset. This extrapolation predicts that two other *Apolemia* species are gelatinous prey specialists like *Apolemia rubriversa*, and predicts that *Erenna laciniata* is a fish specialist like *Erenna richardi*. When predicting soft- and hard-bodied prey specialization, the DAPC achieved 90.9% discrimination success, only marginally confounding hard-bodied specialists with generalists (SM13). The main characters driving this discrimination are involucrum length, heteroneme number, heteroneme volume, tentacle width, total nematocyst volume, total haploneme volume, elastic strand width, and heteroneme length.

## Discussion

### On the evolution of tentillum morphology

The evolutionary history of siphonophore tentilla shows three major transition points which have structured the morphological diversity we see today. First, the earliest split between codonophorans and cystonects divides lineages with penetrating isorhizas (cystonects) from those which utilize heteronemes (codonophorans) for prey capture. Second, the split between apolemiids and eucladophorans divided the simple-tentacled *Apolemia* from the lineage that evolved composite tentilla with heteronemes and haplonemes. Finally, the branch leading to tendiculophorans fostered innovations such as the elastic strands and the terminal filament nematocysts which produced the most complex tentillum structures and greatest morphological diversity we observe among siphonophores.

Siphonophore tentilla are extremely complex and highly diverse. Our analyses show, however, that the siphonophore tentillum morphospace actually has a fairly low extant dimensionality due to having an evolutionary history with many synchronous, correlated changes. This can be due to many causes including structural constraints, developmental constraints, or selection that reduces the viable state space. Though siphonophore development has not been extensively studied, what is known suggests that developmental constraints alone could not explain the highly correlated evolutionary changes we observe. The nematocysts that arm the tentillum are developed in a completely separate region of the gastrozooid (Carré 1972) and then migrate and assemble within the tentillum later on (Skaer 1988). This lack of proximity and physical independence of development between traits makes developmental constraints unlikely. Surprisingly, many of the strong correlations we find are between nematocyst and structural tentillum characters. Therefore, we hypothesize the genetic correlations and phenotypic integration between tentillum and nematocyst characters are maintained through natural selection on separate regulatory networks, out of the necessity to work together and meet the spatial, mechanical, and functional constraints of their prey capture behavior. In order to adequately test these hypotheses, future work would need to study the genetic mechanisms underlying the development of tentilla from a comparative, evolutionary approach. Fortunately, the unique biology of siphonophore tentacles displays the full developmental sequence of tentilla along each tentacle, making siphonophores an ideal system for the comparative study of development.

In Damian-Serrano et al. (2021) we examined the covariance terms in the multivariate rate matrix for the evolution of tentillum and nematocyst characters. Building on this work, here we examine the correlations among the trait values while accounting for phylogenetic structure. The results for both analyses indicate that tentilla are not only phenotypically integrated (with widespread evolutionary correlations across structures) but also show patterns of evolutionary modularity, where different sets of characters appear to evolve in stronger correlations among each other than with other characters (Wagner 1996). This may be indicative of the underlying genetic and developmental dependencies among closely-related nematocyst types and other homologous structures. In addition, these evolutionary modules point to hypothetical functional modules. For example, the coiling degree of the cnidoband and the extent of the involucrum have correlated rates of evolution, while the involucrum may help direct the whiplash of the uncoiling cnidoband distally (toward the prey). The evolutionary innovation of the Tendiculophora tentilla with shooting cnidobands and modular regions may have facilitated further dietary diversification. A specific instance of this dietary diversification may have been the access to abundant small crustacean prey such as copepods. The rapid darting escape response of copepods may preclude their capture in siphonophores without shooting cnidobands. The trophic opportunities unlocked by these morphological novelties may be responsible for the far greater number of species in Tendiculophora than its relatives Cystonectae, Apolemiidae, and Pyrostephidae.

### Heterochrony and convergence in the evolution of tentilla with diet

In addition to identifying shifts in prey type, Damian-Serrano et al. (2021) revealed the specific morphological changes in the prey capture apparatus associated with these shifts. Copepod-specialized diets have evolved independently in *Cordagalma* and some calycophorans. These evolutionary transitions happened together with transitions to smaller tentilla with fewer and smaller cnidoband nematocysts. We found that these morphological transitions evolved convergently in these taxa. Tentilla are expensive single-use structures (Mackie et al. 1987), therefore we would expect that specialization in small prey would beget reductions in the size of the prey capture apparatus to the minimum required for the ecological performance. Such a reduction in size would require extremely fast rates of trait evolution in an ordinary scenario. However, *Cordagalma*’s tentilla strongly resemble the larval tentilla (only found in the first-budded feeding body of the colony) of their sister genus *Forskalia*. This indicates that the evolution of *Cordagalma* tentilla could be a case of paedomorphic heterochrony associated with predatory specialization on smaller prey. This developmental shift may have provided a shortcut for the evolution of a smaller prey capture apparatus.

Our work identifies yet another novel example of convergent evolution. The region of the tentillum morphospace occupied by calycophorans was independently occupied by the physonect *F. vityazi* (Fig. 7B). Like calycophorans, *Frillagalma* tentilla have small C-shaped cnidobands with a few rows of anisorhizas. Unlike calycophorans, they lack paired elongate microbasic mastigophores. Instead, they bear exactly three oval stenoteles, and their cnidobands are followed by a branched vesicle, unique to this genus. Their tentillum morphology is very different from that of other related physonects, which tend to have long, coiled, cnidobands with many paired oval stenoteles. Our SURFACE analysis clearly indicates a regime convergence in the cnidoband morphospace between *Frillagalma* and calycophorans (Fig. 7B). Most studies on calycophoran diets have reported their prey to consist primarily of small crustaceans, such as copepods or ostracods (Purcell 1981, 1984). The diet of *F. vityazi* is unknown, but this morphological convergence suggests that they evolved to capture similar kinds of prey. However, our DAPCs predict that *Frillagalma* has a generalist niche (Fig. 10) with both soft and hard-bodied prey (SM13).

### Evolution of nematocyst shape

A remarkable feature of siphonophore haplonemes is that they are outliers to all other Medusozoa in their surface area to volume relationships, deviating significantly from sphericity (Thomason 1988). This suggests a different mechanism for their discharge that could be more reliant on capsule tension than on osmotic potentials (Carré and Carré 1980), and strong selection for efficient nematocyst packing in the cnidoband (Skaer 1988; Thomason 1988). Our results show that Codonophora underwent a shift toward elongation and Cystonectae toward sphericity, assuming the common ancestor had an intermediate state. Since we know that the haplonemes of other hydrozoan outgroups are generally spheroidal, it is more parsimonious to assume that cystonects are simply retaining this ancestral state. We observe a return to more rounded (ancestral) haplonemes in *Erenna*, concurrent with a secondary gain of a piscivorous trophic niche, like that exhibited by cystonects. Our SURFACE analysis shows that this transition to roundness is convergent with the regime occupied by cystonects (Fig. 7A). Purcell (1984) showed that haplonemes have a penetrating function as isorhizas in cystonects and an adhesive function as anisorhizas in Tendiculophora. It is no coincidence that the two clades that have converged to feed primarily on fish have also converged morphologically toward more compact haplonemes. Isorhizas in cystonects are known to penetrate the skin of fish during prey capture, and to deliver the toxins that aid in paralysis and digestion (Hessinger 1988). *Erenna*’s anisorhizas are also able to penetrate human skin and deliver a painful sting (Pugh 2001), a common feature of piscivorous cnidarians like the Portuguese man-o-war or box jellies.

The implications of these results for the evolution of nematocyst function are that an innovation in the discharge mechanism of haplonemes may have occurred during the main shift to elongation. Elongate nematocysts can be tightly packed into cnidobands. We hypothesize this may be a Tendiculophora lineage-specific adaptation to packing more nematocysts into a limited tentillum space, as suggested by (Skaer 1988). Thomason (1988) hypothesized that smaller, more spherical nematocysts, with a lower surface area to volume ratio, are more efficient in osmotic-driven discharge and thus have more power for skin penetration. The elongated haplonemes of crustacean-eating Tendiculophora have never been observed penetrating their crustacean prey (Purcell 1984), and are hypothesized to entangle the prey through adhesion of the abundant spines to the exoskeletal surfaces and appendages. Entangling requires less acceleration and power during discharge than penetration, as it does not rely on point pressure. In fish-eating cystonects and *Erenna* species, the haplonemes are much less elongated and very effective at penetration, in congruence with the osmotic discharge hypothesis. Tendiculophora, composed of the clades Euphysonectae and Calycophorae, includes the majority of siphonophore species. Within these clades are the most abundant siphonophore species, and a greater morphological and ecological diversity is found. We hypothesize that this packing-efficient haploneme morphology may have also been a key innovation leading to the diversification of this clade. However, other characters that shifted concurrently in the lineage leading to this clade could have been equally responsible for their extant diversity.

All cnidarians are characterized by bearing nematocysts used primarily for defense and prey capture. The patterns we revealed in siphonophores may reflect more general patterns in the evolution of nematocysts across cnidarians. Siphonophore tentilla are unique in many ways, but also bear similarities to other structures found in other cnidarians. For example, many anemones bear specialized, nematocyst-laden filaments named acontia, which they use for defense and territorial competition (Shick 2012). These filaments also carry tightly packed, extremely elongated nematocysts (mastigophores and isorhizas). This extreme elongation may have also arisen as an adaptation to pack a higher number of nematocysts in a small space. While siphonophore nematocyst elongation may be an outlier among Medusozoa, similar morphologies can be commonly found across Actiniaria and Hexacorallia. These morphological shifts may also involve changes to the discharge mechanisms and nematocyst function. Answering this question requires further research on the discharge mechanics of nematocysts beyond model organisms like *Hydra*. As shown in Fig. 3, siphonophores bear a large variety of nematocyst types and subtypes. Different heteroneme subtypes vary widely in shaft and filament complexity, ranging from the simplest mastigophores to three-spined stenoteles or double-bulged birhopaloids. However, the functional differences between these subtypes are still poorly known. Further research is necessary to fully comprehend the evolutionary and ecological implications of these transitions in nematocyst subtype.

### Generating hypotheses on siphonophore feeding ecology

One motivation for our research is to understand the links between prey-capture tools and diets, so we can generate hypotheses about the diets of predators based on morphological characteristics. Indeed, our discriminant analyses were able to distinguish between different siphonophore diets based on morphological characters alone. The models produced by these analyses generated testable predictions about the diets of many species for which we only have morphological data of their tentacles. For example, the unique tentilla morphology of *Frillagalma* is predicted to render a generalist diet, or one of the undescribed deep-sea physonect species examined is predicted to be a fish specialist, which is congruent with its close phylogenetic relationship to other piscivorous physonects. While the limited dataset used here is informative for generating tentative hypotheses, empirical dietary data are still scarce and insufficient to cast robust predictions. In future work, we will test these ecological hypotheses and validate these models by directly characterizing the diets and feeding habits of some of those siphonophore species. Predicting diet using morphology is a powerful tool to reconstruct food web topologies from community composition alone. In many of the ecological models found in the literature, interactions among the oceanic zooplankton have been treated as a black box (Mitra 2009). The ability to predict such interactions, including those of siphonophores and their prey, will enhance the taxonomic resolution of nutrient-flow models constructed from plankton community composition data.

## Supplementary Material

obab019_Supplementary_Data
