# Supplementary material for: The Evolutionary History of Siphonophore Tentilla: Novelties, Convergence, and Integration
Source: Integr Org Biol. 2021 May 26;3(1):obab019. doi: 10.1093/iob/obab019 (PMC8331849; doi:10.1093/iob/obab019)
Supplement: obab019_Supplementary_Data [file obab019_supplementary_data.zip › Organismal_supplement_postreview.pdf]

Supplementary Materials

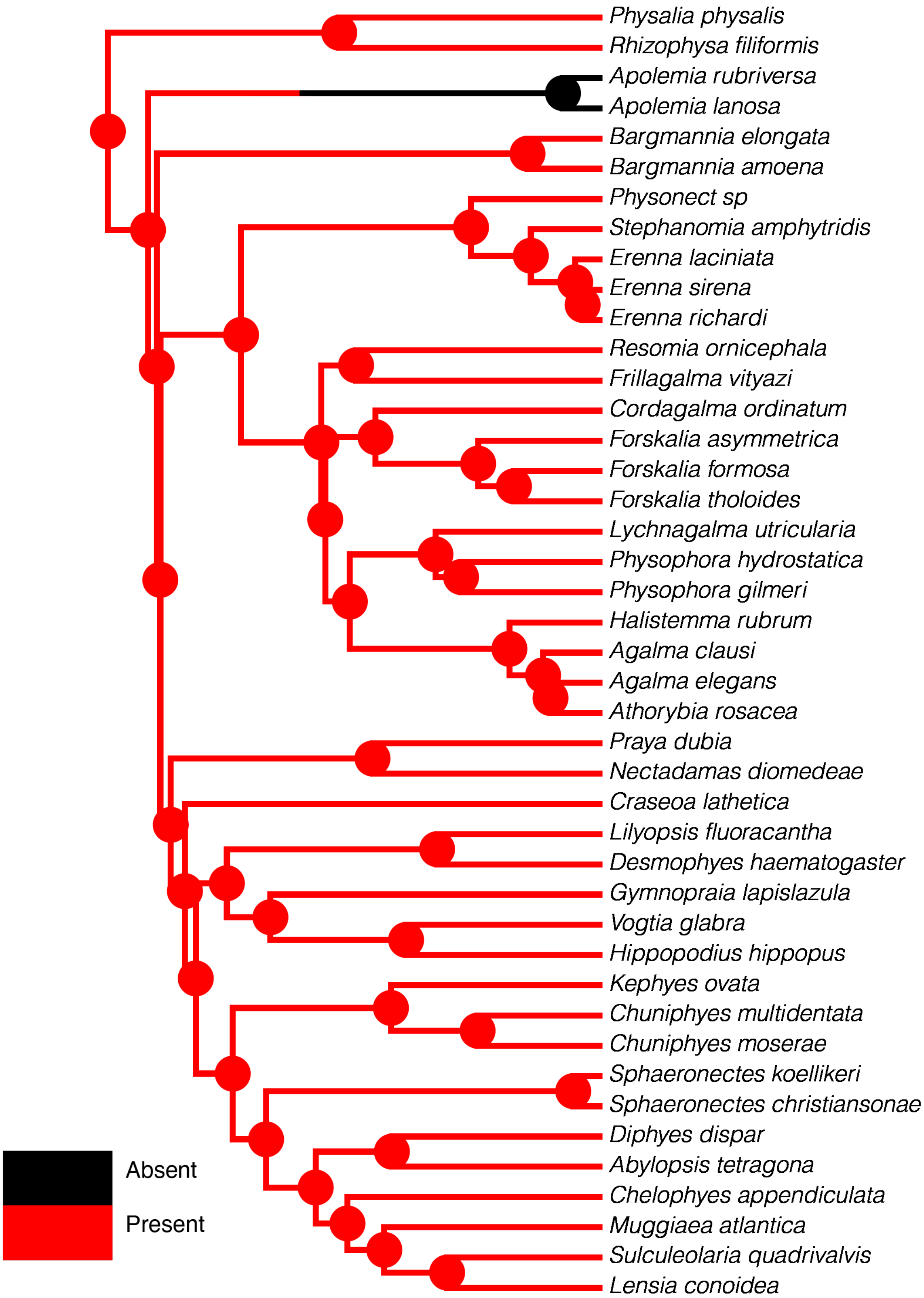

Figure 1: SIMMAP Tentilla presence/absence.

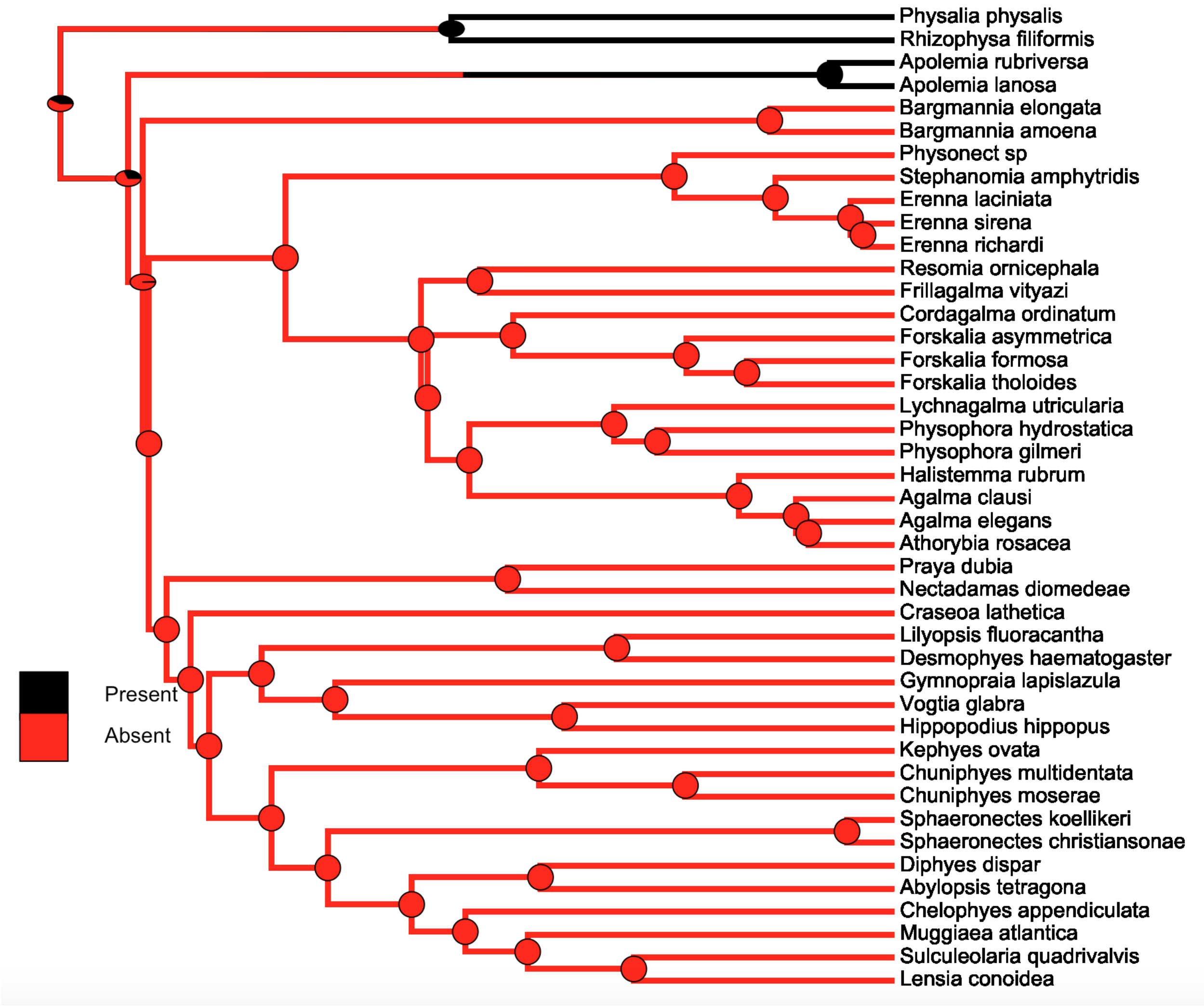

Figure 2: SIMMAP Cnidoband proximal heteroneme presence/absence.

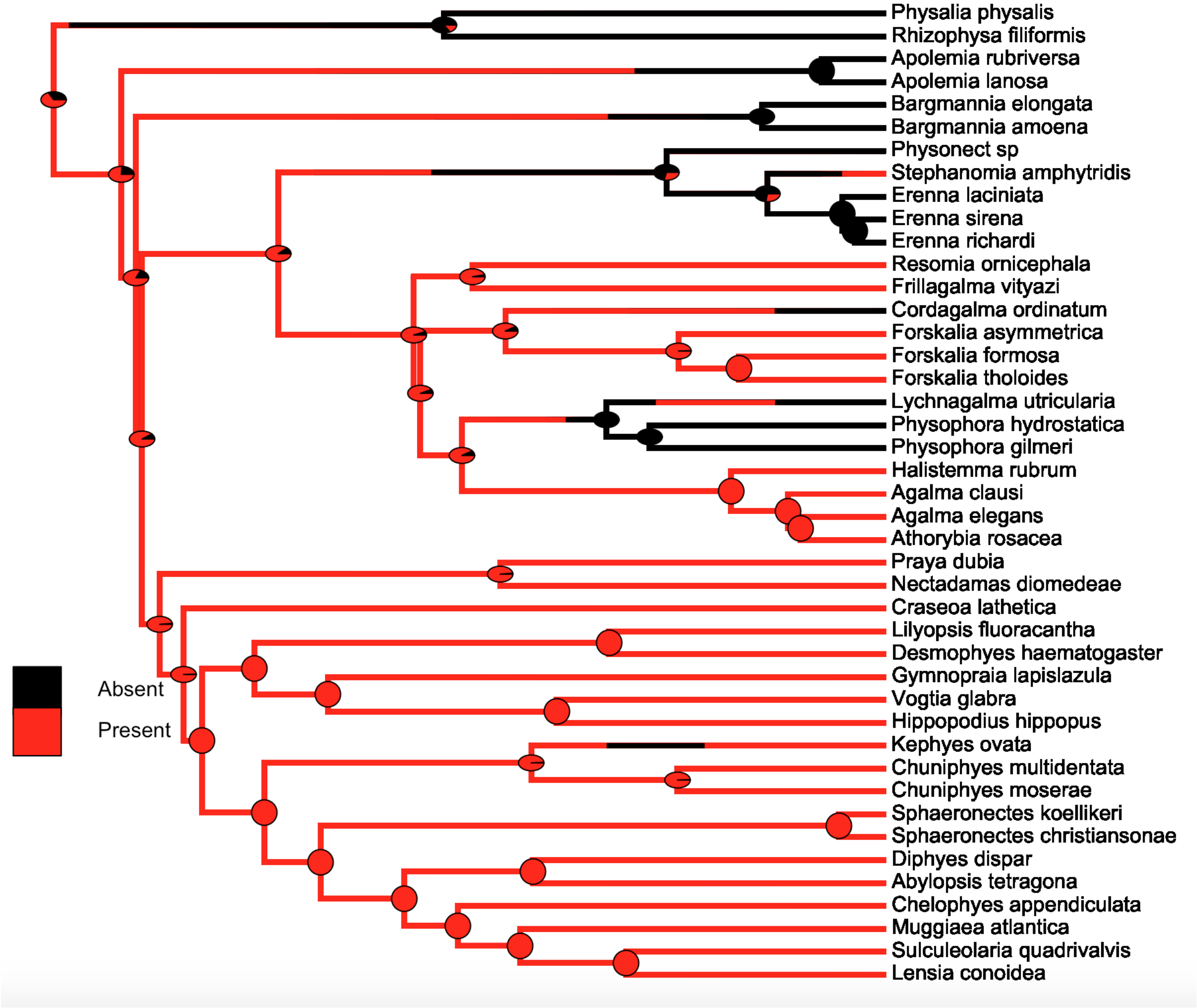

Figure 3: SIMMAP Desmoneme+Rhopaloneme presence/absence.

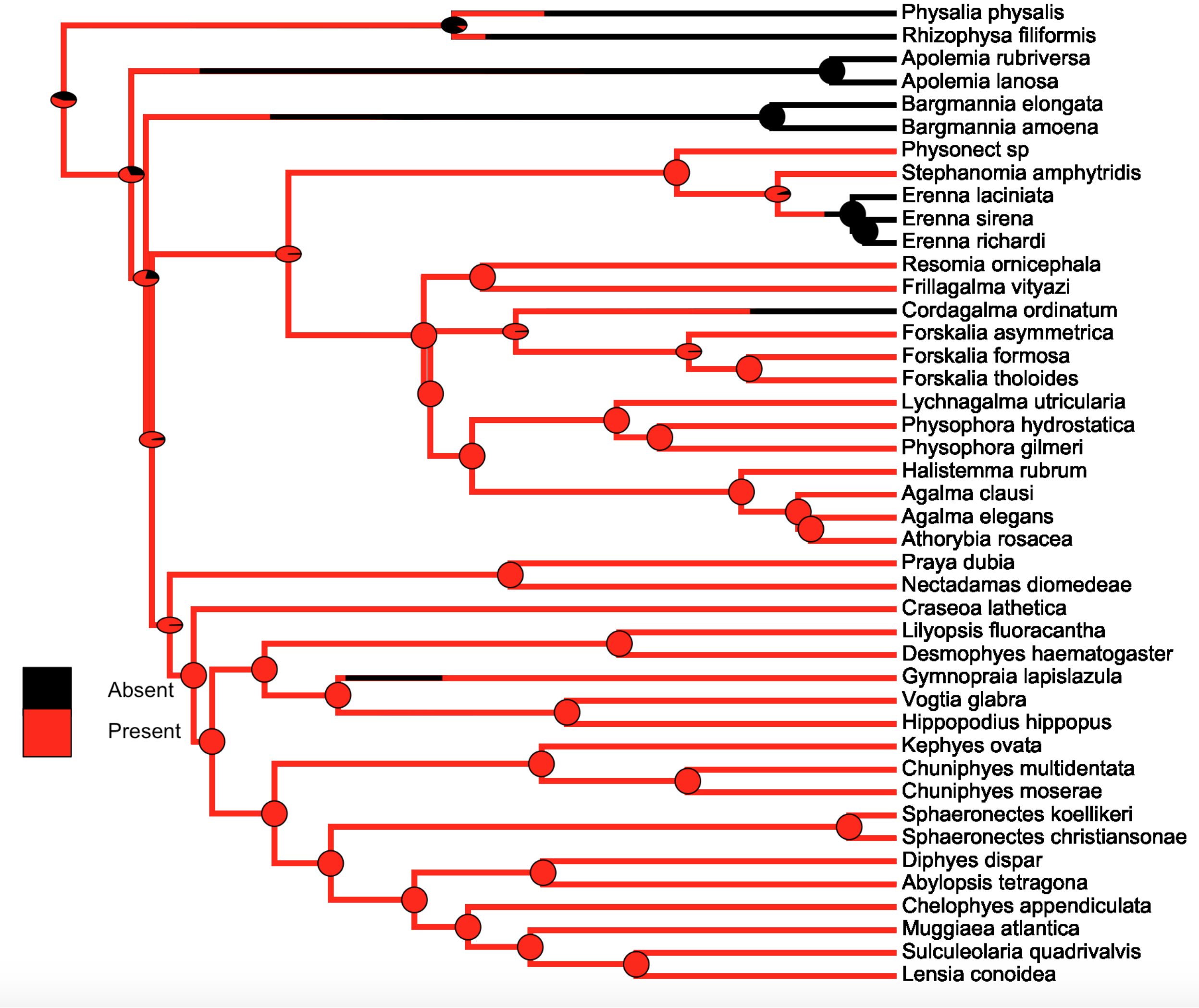

Figure 4: SIMMAP Actively discharging cnidobands presence/absence.

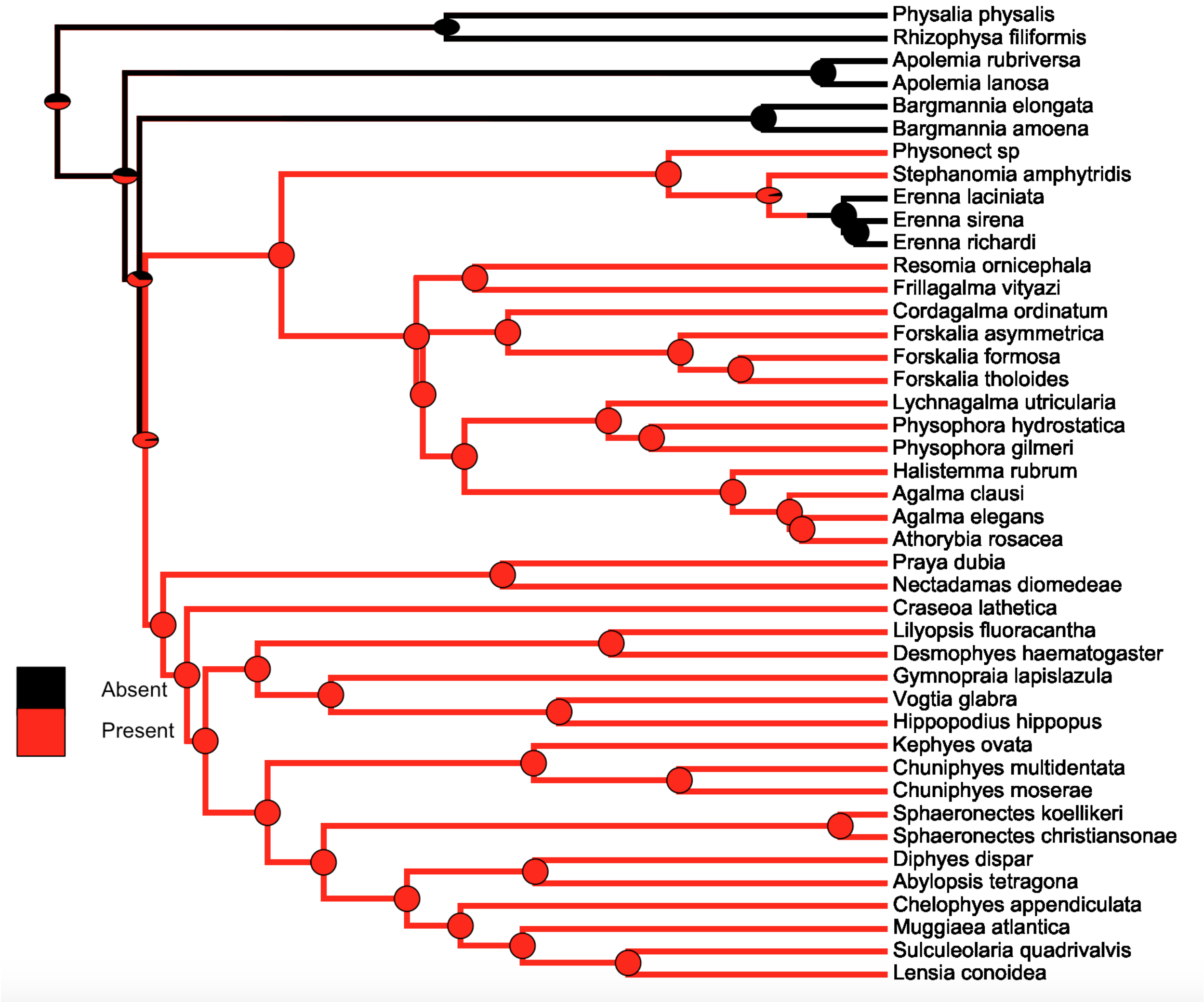

Figure 5: SIMMAP Elastic strands presence/absence.

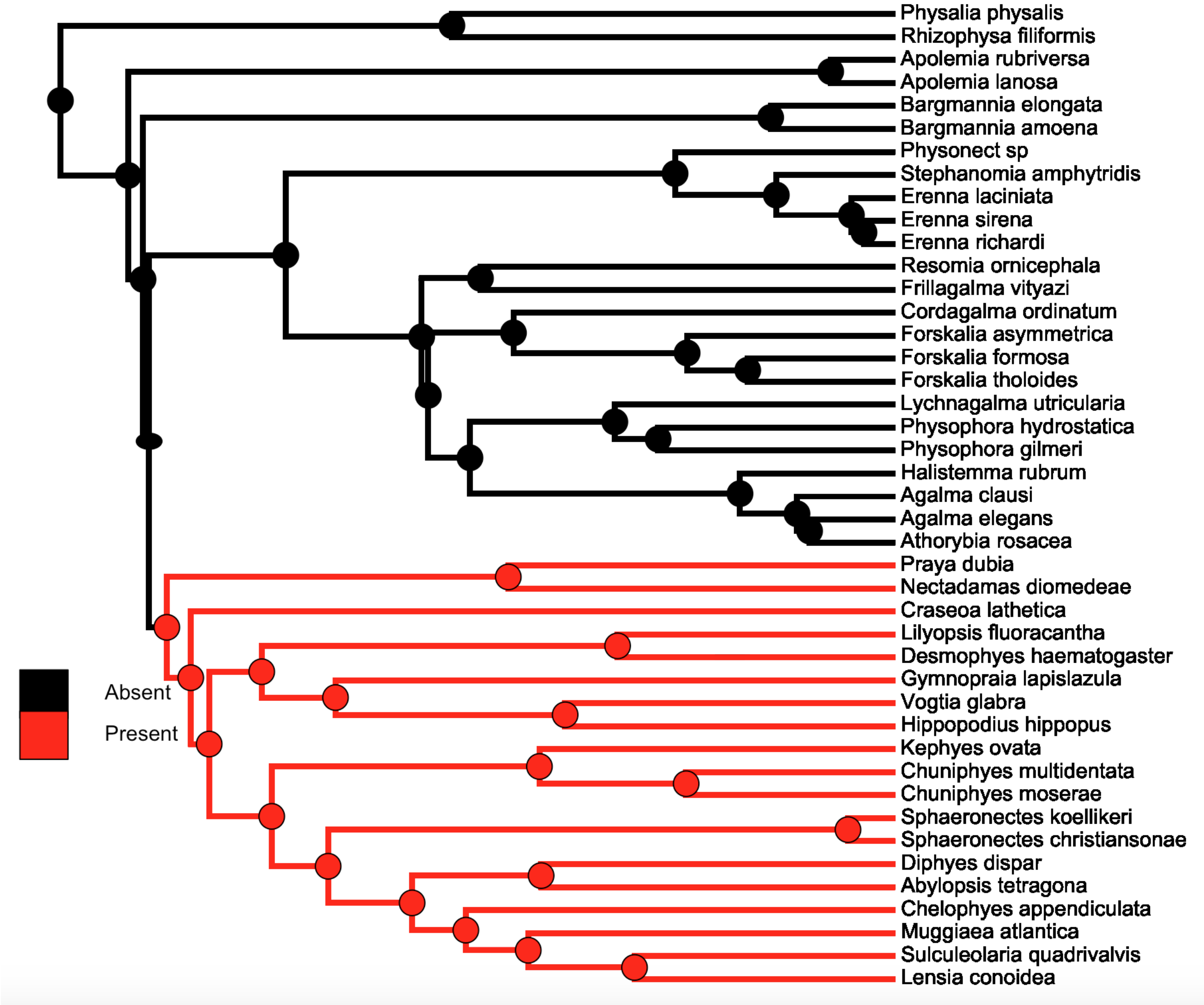

Figure 6: SIMMAP Cnidoband distal desmonemes presence/absence.

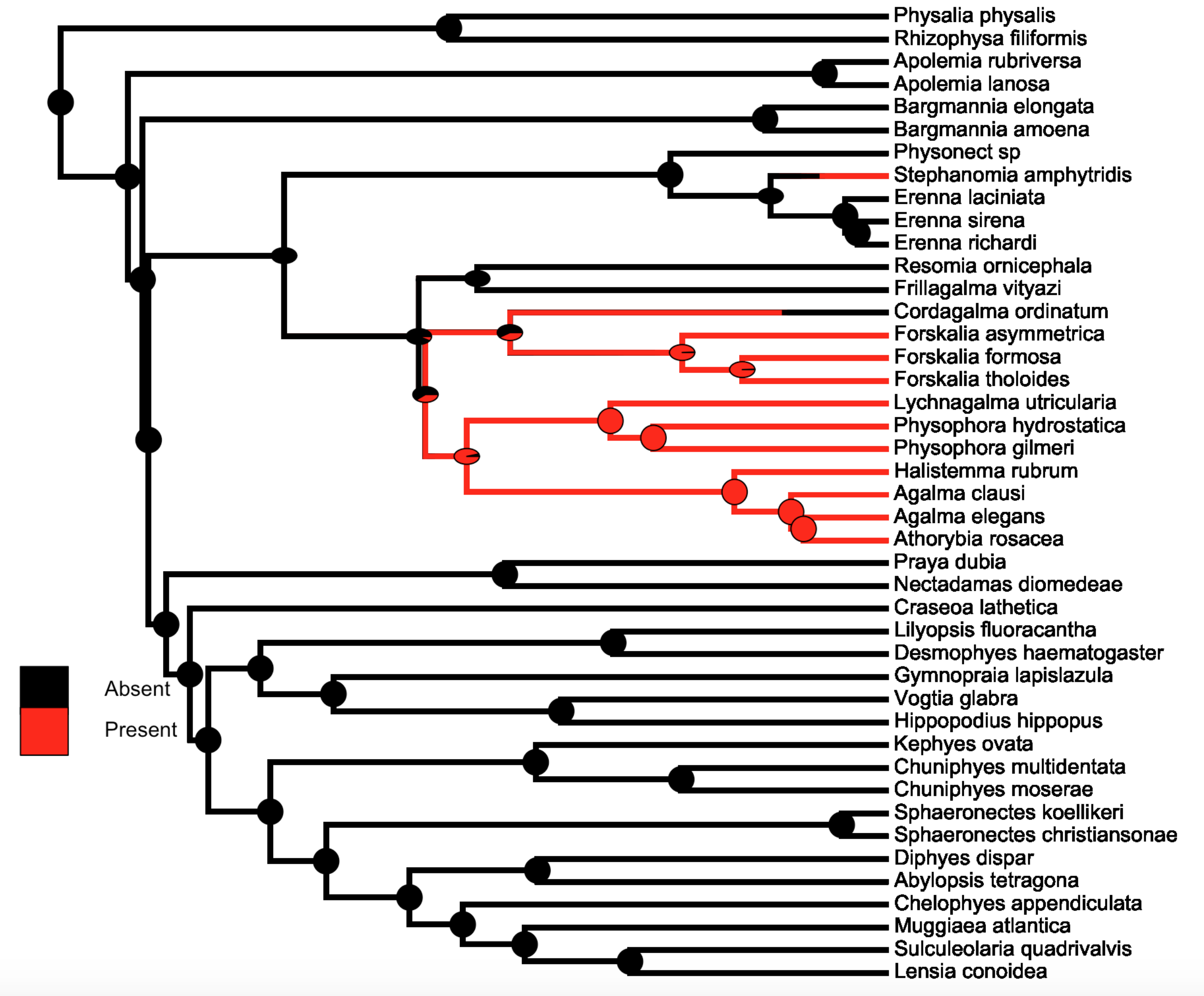

Figure 7: SIMMAP Coiled cnidoband phenotype presence/absence.

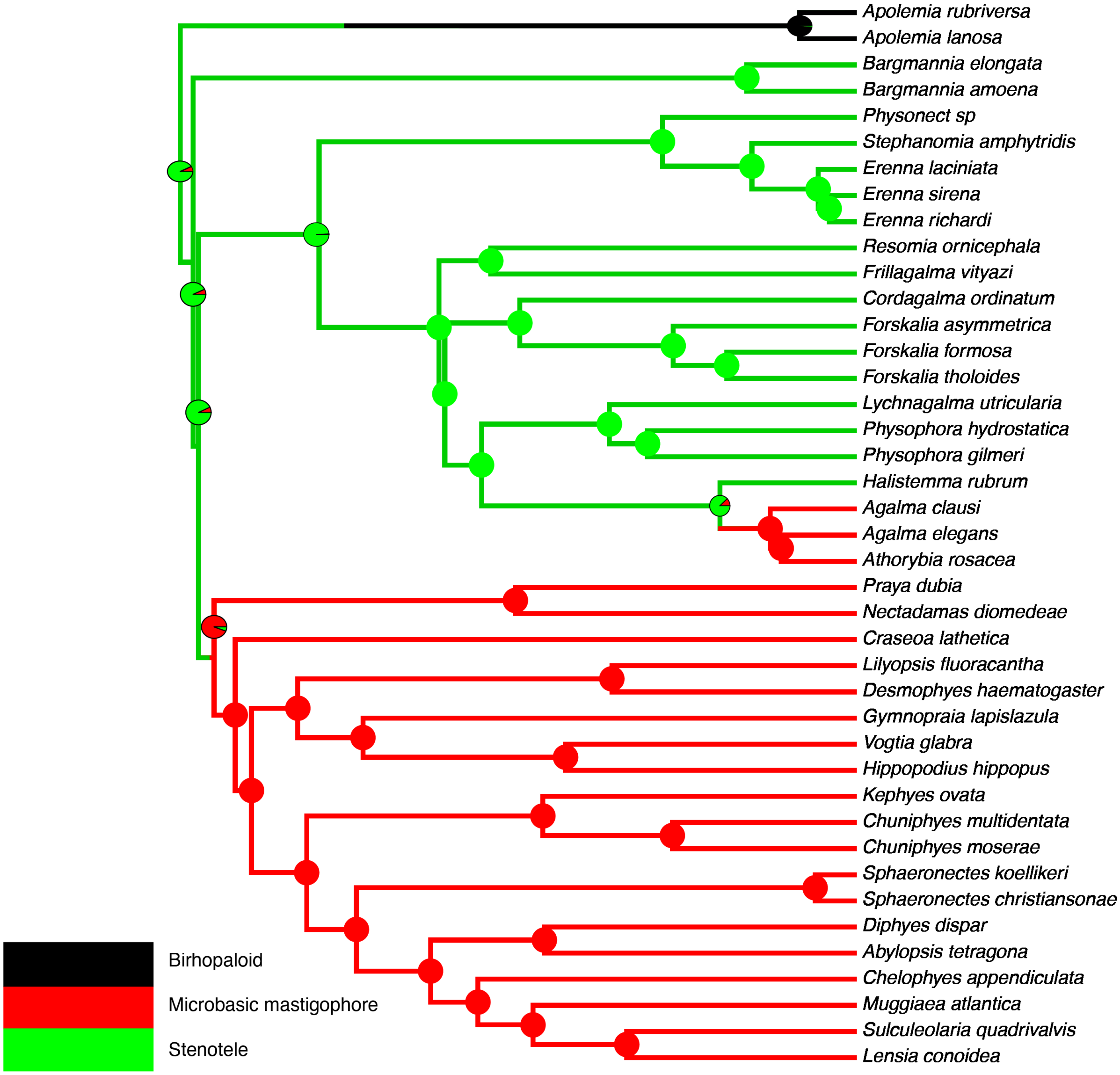

Figure 8: SIMMAP Heteroneme type.

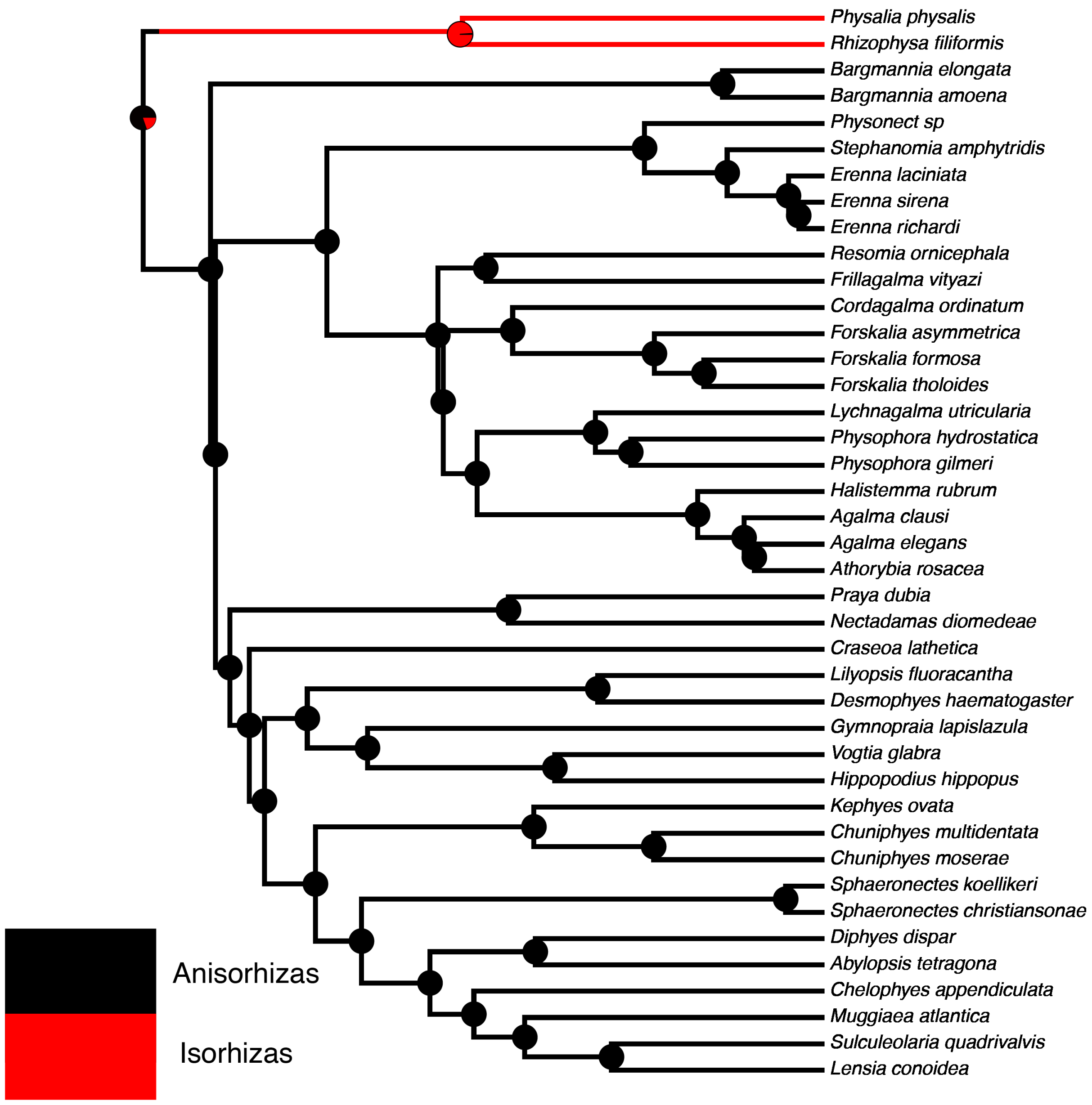

Figure 9: SIMMAP Haploneme type.

Character	Non-Phylogenetic dAIC	BM dAIC	EB dAIC	OU dAIC	K	K p-value	Ntaxa
**Haploneme elongation**	0	2.017	4.332	2.38	0.583	0.001	43
**Desmoneme elongation**	0	3.232	5.693	3.183	0.018	0.864	31
**Heteroneme shaft width µm**	0	5.346	7.67	2.581	0.45	0.005	42
**Elastic strand width µm**	0	1526	3.938	1.296	0.706	0.001	34
**Desmoneme length µm**	0.518	0	2.46	0.578	0.566	0.002	31
**Heteroneme shaft extension**	0.589	0	2.324	1.965	0.041	0.970	42
**Haploneme SA/V**	0.91	0	2.315	2.291	0.156	0.132	43
**Total heteroneme volume µm3**	0.961	0	2.352	2.328	0.248	0.046	39
**Rhopaloneme width µm**	1.205	0	2.46	1.321	0.308	0.123	31
**Heteroneme volume µm3**	2.002	0	2.153	2.324	0.196	0.117	42
**Involucrum length µm**	2.479	0	2.498	2.492	0.529	0.001	29
**Tentacle width µm**	2.939	0	2.307	1.974	0.367	0.044	44
**Cnidoband coiledness**	3.077	0	2.315	1.786	0.174	0.043	43
**Total haploneme volume µm3**	3.641	0	1.852	2.296	0.198	0.267	43
**Cnidoband free length µm**	3.801	0	2.132	2.315	0.325	0.007	43
**Heteroneme free length µm**	3.82	0	2.01	1.325	0.301	0.080	42
**Rhopaloneme elongation**	3.852	0	2.145	2.46	0.062	0.827	31
**Desmoneme width µm**	3.96	0	2.46	2.121	0.553	0.004	31
**Cnidoband length µm**	4.094	0	1.911	2.315	0.321	0.015	43
**Heteroneme number**	4.262	0	2.352	2.219	0.866	0.001	39
**Heteroneme shaft free length µm**	4.553	0	2.324	2.321	0.331	0.126	42
**Rhopaloneme length µm**	5.599	0	2.46	2.457	0.589	0.001	31
**Heteroneme/Cnidoband length**	5.671	0	1.862	2.342	1.068	0.001	42
**Pedicle width µm**	6.566	0	2.253	2.315	0.541	0.001	43
**Haploneme width µm**	7.495	0	2.218	2.304	0.553	0.001	43
**Heteroneme width µm**	7.53	0	2.324	1.647	0.502	0.001	42
**Heteroneme elongation**	14.169	0	0.819	2.23	0.508	0.001	42
**Haploneme row number**	19.566	0	2.114	2.315	0.442	0.001	43
**Total nematocyst volume µm3**	21.007	0	2.213	2.292	1.3	0.001	45
**Cnidoband width µm**	5.69	0.307	0	2.623	0.374	0.001	43
**Haploneme free length µm**	12.337	7.125	0	9.439	1.079	0.001	43

Non-phylogenetic model supported

Brownian Motion model supported

Early Burst model supported

Figure 10: Model support (delta AICc), phylogenetic signal (Blomberg's K), and phylogenetic signal permutation test p-value for each continuous character. Ntaxa = Number of taxa used in the analyses after removing those where the character sate is inapplicable or the data is missing.

Variable	Best model	Msig	Cvar	Svar	Sasr	Shgt	Dcfd
Desmoneme length µm	WN	0.889	0.224	0.084	0.32	0.146	0
Heteroneme shaft extension	WN	0.861	0	0.577	0	0.533	0.042
Total heteroneme volume	WN	0.895	0.577	0.006	0.026	0.078	0.603
Rhopaloneme width µm	WN	0.823	0.42	0.182	0.014	0.531	0.006
Haploneme free length µm	EB	0.841	0.052	0.036	0.168	0.226	0.843
Heteroneme volume µm3	BM	0.855	0.731	0.228	0.897	0.775	0.104
Involucrum length µm	BM	0.839	0.01	0.018	0.116	0.09	0.987
Tentacle width µm	BM	0.817	0.841	0.402	0.386	0.785	0.48
Cnidoband coiledness	BM	0.873	0	0.028	0.016	0.144	0.41
Total haploneme volume	BM	0.807	0.228	0.004	0.006	0.024	0.398
Cnidoband free length µm	BM	0.825	0.076	0.002	0	0.006	0.681
Heteroneme free length µm	BM	0.859	0.392	0.386	0.056	0.591	0.284
Rhopaloneme elongation	BM	0.873	0.022	0.006	0.004	0.048	0.104
Desmoneme width µm	BM	0.813	0.877	0.531	0.014	0.941	0.014
Cnidoband length µm	BM	0.829	0.096	0	0	0.004	0.901
Heteroneme nµmber	BM	0.823	0.312	0	0.004	0.02	0.869
Heteroneme shaft free length µm	BM	0.877	0.468	0.565	0.034	0.841	0.851
Rhopaloneme length µm	BM	0.829	0.525	0.547	0.01	0.917	0.08
Heteroneme/cnidoband length	BM	0.839	0.01	0	0.004	0.008	0.715
Cnidoband width µm	BM	0.907	0.977	0	0.002	0.01	0.11
Pedicle width µm	BM	0.817	0.931	0.476	0.088	0.969	0.813
Haploneme width µm	BM	0.881	0.805	0.12	0.294	0.511	0.15
Heteroneme width µm	BM	0.849	0.142	0.156	0.356	0.819	0.278
Heteroneme elongation	BM	0.933	0.094	0.07	0.681	0.791	0.777
Haploneme row number	BM	0.863	0	0.002	0.004	0.008	0.012
Total nematocyst volume	BM	0.809	0.521	0.024	0.016	0.198	0.837
Haploneme surface: volume	BM	0.831	0.945	0.130	0.503	0.426	0.170

Figure 11: P-values of the model adequacy score tests for the best model supported for each morphological character. Cvar = coefficient of variation of the absolute value of the contrasts. Svar = Slope of a linear model fitted to the absolute value of the contrasts against their expected variances. Sasr = slope of the contrasts against the ancestral state inferred at each corresponding node. Shgt = slope of the contrasts against node depth. Dcfd = Kolmolgorov-Smirnov D-statistic comparing contrasts to a normal distribution with SD equal to the root of the mean of squared contrasts. P-values < 0.05 were highlighted in grey, indicating significant deviations between the model and the observed data.

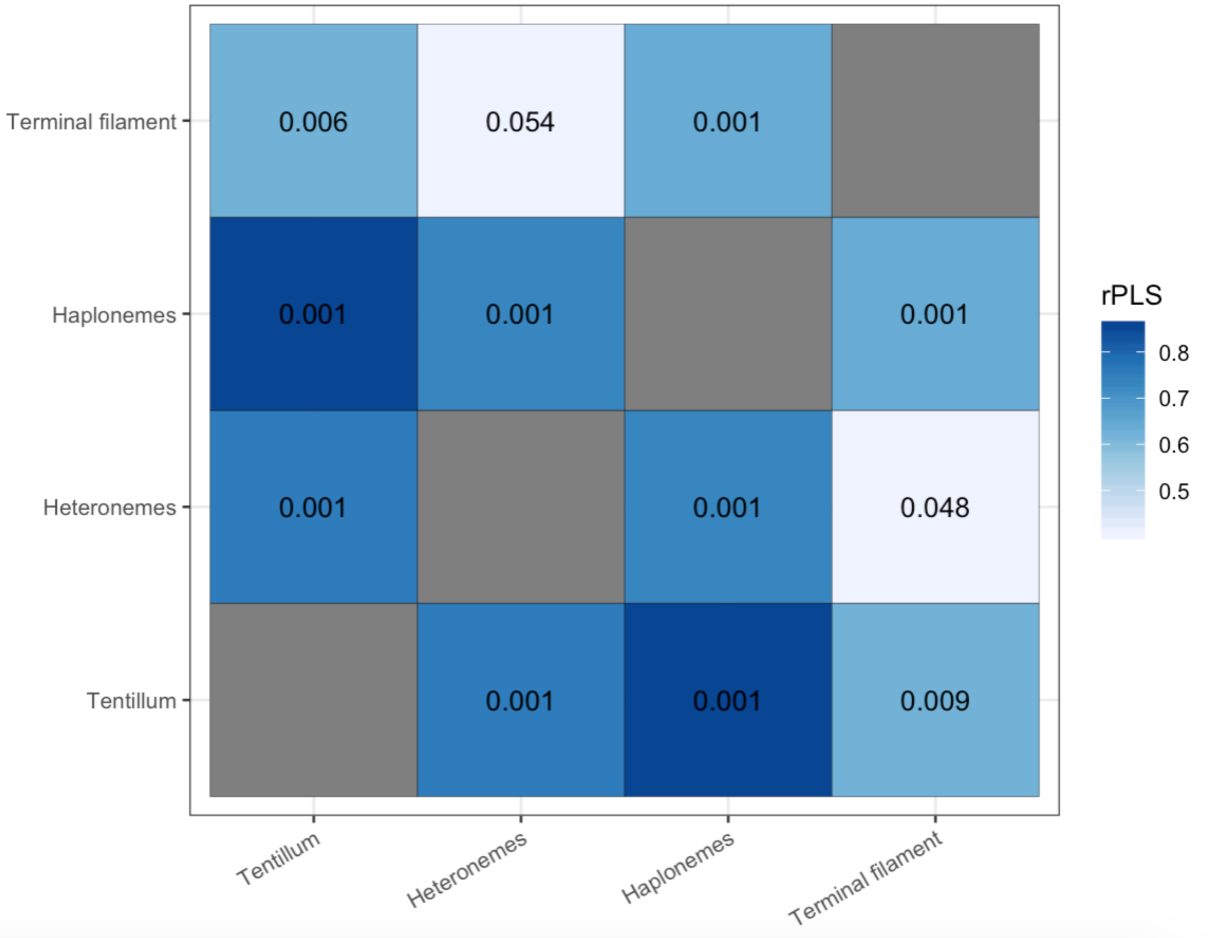

Figure 12: Heatmap showing the phenotypic integration between character modules accounting for phylogeny. Text in cells shows p-values. Color indicates the partial least squares (PLS) multivariate correlation coefficients.

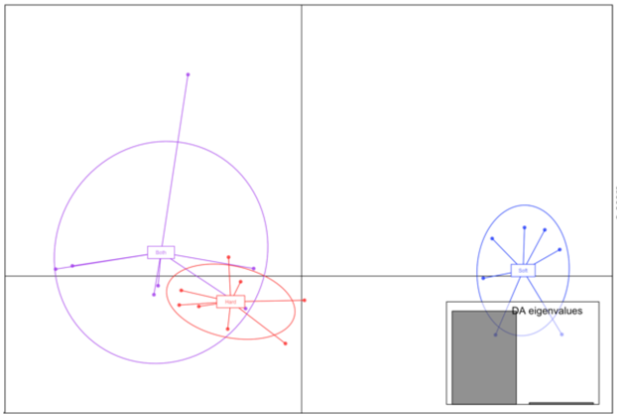

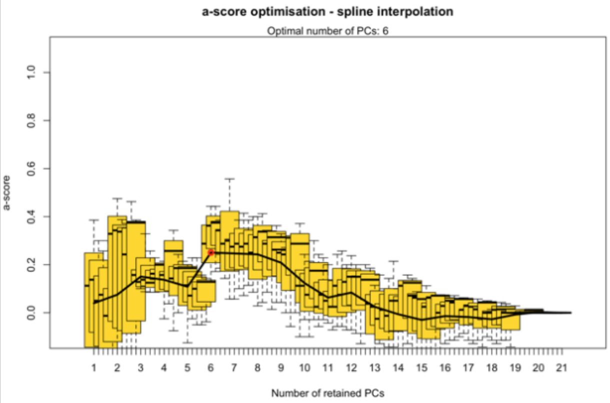

Variable contribution

Involucrum.length..um.	24.425696
Heteroneme.number	18.129947
Heteroneme.volume..um3.	6.849738
Tentacle.width..um.	6.587487
Total_nematocyst_volume	5.606488
total_haploneme_volume	4.185115
Elastic.strand.width..um.	3.584917
Heteroneme.free.length..um.	3.014292

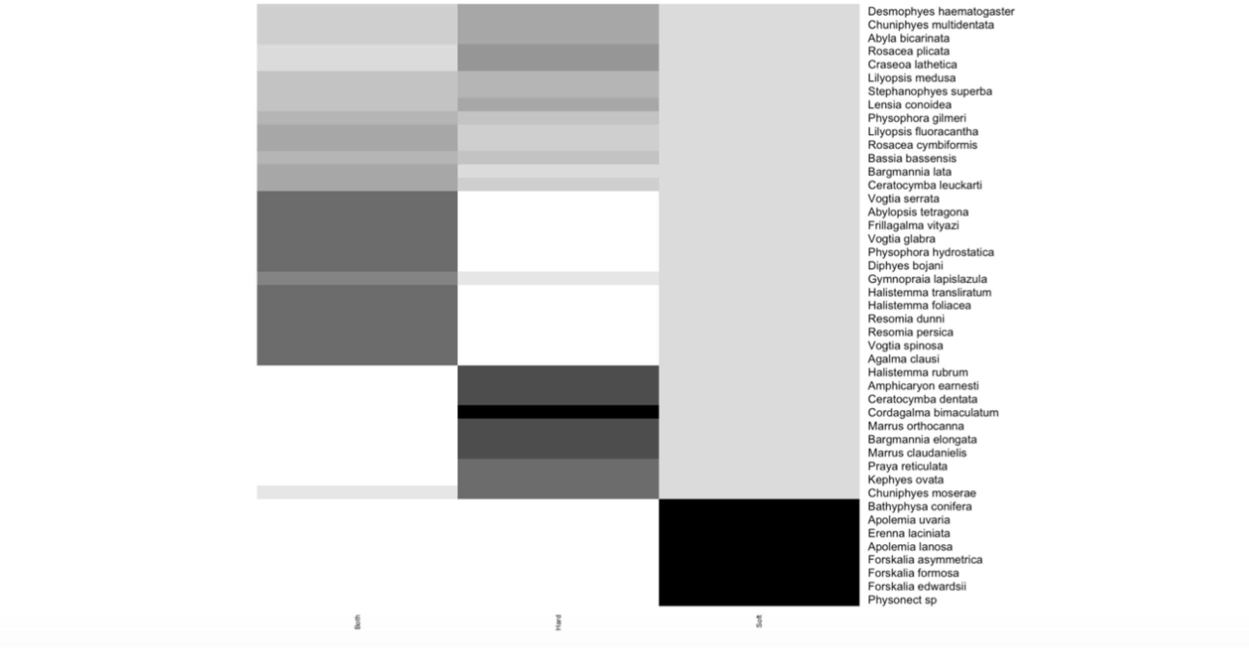

Figure 13: DAPC for soft-bodied vs. hard bodied prey specialization. Six PCs retained after a-score optimization (100 iterations). Two LDA functions used. Discriminant power on training set: 90.9%. Grayscale heat map shows the posterior probability distribution of the predictions. Variable contribution (top quartile) calculated by the sum of the LDA variable loadings weighted by the eigenvalue of each LDA.

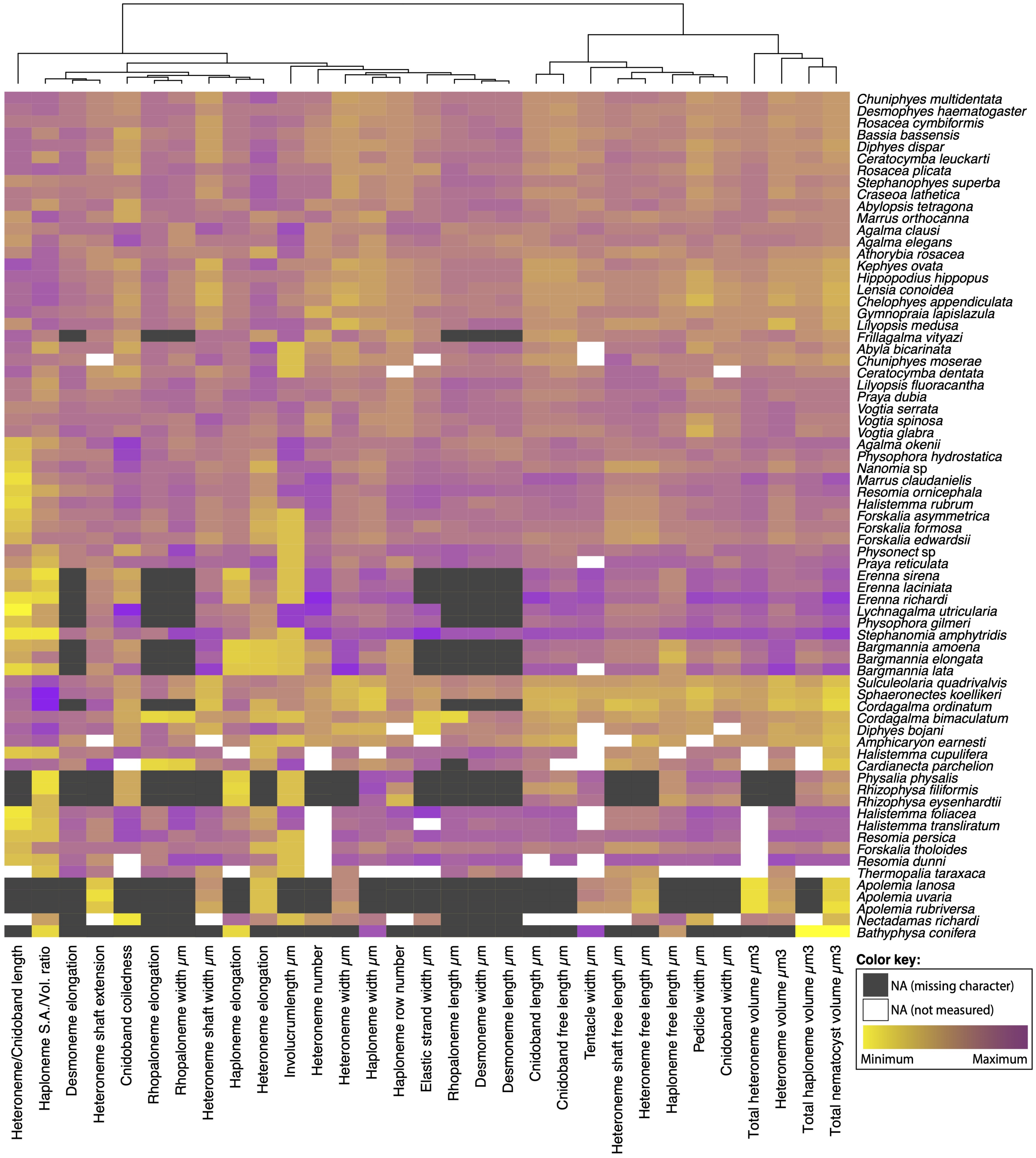

Figure 14: Heatmap summarizing the morphological diversity measured in Damian-Serrano *et al.* 2020 for 96 species of siphonophores clustered by similarity (raw data published in Damian-Serrano 2020). Missing values from absent characters presented as dark grey cells, missing values produced from technical difficulties presented as white cells. Values scaled by character.

S1.1) Definitions of homologous structures used throughout this work.

Structure	Definition
**Haploneme**	Nematocyst with no shaft
**Heteroneme**	Nematocyst with a distinct shaft
**Desmoneme**	Small oval/tapered adhesive nematocyst with thick coiled tubule
**Rhopaloneme**	Small rod-like nematocyst found on the terminal filament
**Terminal filament**	Distal extension of the tentillum beyond the cnidoband
**Cnidoband**	Distinct packing of nematocysts on the dorsal side of the tentillum
**Tentacle**	Tubular projection from the gastrozooid basigaster
**Tentillum**	Evenly spaced dorsal evagination of the tentacle carrying ordered and functional nematocysts
**Involucrum**	Extension of the pedicle covering part of the cnidoband
**Pedicle**	Proximal region of the tentillum between the cnidoband and the tentacle
**Elastic strand**	Mesoglea derived collagenous double strand underlying the cnidoband of some siphonophores

S1.2) Definitions of the continuous morphological and kinematic characters measured.

Character	Definition	Units
**Cnidoband length**	Distance from the base to the tip of the cnidoband in natural position	micrometers
**Cnidoband free length**	Distance from the base to the tip of the cnidoband when stretched straight	micrometers
**Cnidoband width**	Diameter of the cnidoband on the widest point	micrometers
**Involucrum length**	Length of the involucrum from the base of the cnidoband to its most distal extent	micrometers
**Heteroneme length**	Length of the heteronemes	micrometers
**Heteroneme width**	Diameter of the heteronemes at the widest point	micrometers
**Heteroneme shaft length**	Length of the heteroneme shaft	micrometers
**Heteroneme shaft width**	Width of the heteroneme shaft	micrometers
**Heteroneme number**	Number of heteronemes in each tentillum (# in each row*2)	micrometers
**Haploneme length**	Length of the haplonemes	micrometers
**Haploneme width**	Diameter of the haplonemes at the widest point	micrometers
**Rhopaloneme length**	Length of the rhopalonemes	micrometers
**Rhopaloneme width**	Diameter of the rhopalonemes at the widest point	micrometers
**Desmoneme length**	Length of the desmonemes	micrometers
**Desmoneme width**	Diameter of the cnidoband at the widest point	micrometers
**Involucrum length**	Length of the involucrum from the base of the cnidoband to its most distal extent	micrometers
**Elastic strand width**	Diameter of the descending elastic strand at the widest point	micrometers
**Pedicle width**	Diameter of the pedicle	micrometers
**Tentacle width**	Diameter of the tentacle	micrometers
**Haploneme row number**	Number of haploneme rows running parallel to the length of the cnidoband	micrometers
**Cnidoband coiledness**	Cnidoband free length / Cnidoband length	adimensional
**Heteroneme elongation**	Heteroneme Length/Width	adimensional
**Haploneme elongation**	Haploneme Length/Width	adimensional
**Desmoneme elongation**	Desmoneme Length/Width	adimensional
**Rhopaloneme elongation**	Rhopaloneme Length/Width	adimensional
**Heteroneme shaft extension**	Heteroneme shaft length / Heteroneme capsule length	adimensional
**Nematocyst Surface area**	4*pi*(2*((((Length/2)*(Width/2))^1.6)+(((Width/2)^2)^1.6))/3)^(1/1.6)	micrometers squared
**Nematocyst volume**	Ellipsoid formula : (4/3)*pi*(Length/2)*((Width/2)^2)	micrometers cubed
**Nematocyst SA/V ratio**	Nematocyst surface area / Nematocyst volume	1/micrometers
**Total haploneme volume**	Haploneme volume * Haploneme row number * (Cnidoband free length / Haploneme width)	micrometers cubed
**Total heteroneme volume**	Heteroneme volume * Heteroneme number	micrometers cubed
**Total nematocyst volume**	Total haploneme volume + Total heteroneme volume	micrometers cubed

Figure 15: Character definitions.
