# Supplementary material for: The Evolutionary History of Siphonophore Tentilla: Novelties, Convergence, and Integration
Source: Integr Org Biol. 2021 May 26;3(1):obab019. doi: 10.1093/iob/obab019 (PMC8331849; doi:10.1093/iob/obab019)
Supplement: obab019_Supplementary_Data [file obab019_supplementary_data.zip › SM16-slides_catalog_measured.pdf]

Slide	Species	Specimen	Accesion #	Catalog #
A1	Praya dubia	JSL I 1003-S8	YPM.12903	106576
A2	Bargmannia lata	D555-SS1	YPM.12903	106577
A3	Diphyes dispar	RI8-27	YPM.12903	106578
A4	Thermopalia taraxaca	Alvin	YPM.12903	106579
A6	Nanomia bijuga	VF2016-6	YPM.12903	106580
A7	Rhizophysa filiformis	BWP 566-16	YPM.12903	106581
A8	Lensia conoidea	JSL I 2953-SS11	YPM.12903	106582
A9	Apolemia rubriversa	D558-D4	YPM.12903	106583
A10	Nanomia cara	BWP 1420-11	YPM.12903	106584
A11	Nanomia bijuga	D1023-SS2	YPM.12738	106585
A12	Lilyopsis fluoracantha	M122-SS1	YPM.12903	106586
A13	Bathyphysa conifera	JSL I 2657-DS1	YPM.12903	106587
B1	Hippopodius hippopus	VF#6	YPM.12903	106588
B2	Physophora hydrostatica	1845 - 5	YPM.12903	106589
B3	Stephanomia amphytridis	JSL I 2888-CG6 1/2	YPM.12903	106590
B6	Forskalia edwardsii	BWP 542-15	YPM.12903	106591
B7	Rhizophysa filiformis	BWP 588-21	YPM.12903	106592
B8	Lensia conoidea	JSL I 2941-SS11	YPM.12903	106593
B9	Agalma okenii	BWP 786-19	YPM.12903	106594
B10	Lensia conoidea	D499-SS4	YPM.12903	106595
B11	Apolemia lanosa	D1024-D1	YPM.12738	106596
B12	Physophora gilmeri	KM2018-Trawl1	YPM.12939	106597
C3	Chuniphyes multidentata	D494-SS8	YPM.12903	106598
C6	Forskalia edwardsii	BWP 529 For39	YPM.12903	106599
C7	Lychnagalma utricularia	JSL II 990-SS1	YPM.12903	106600
C8	Lensia conoidea	JSL I 2935-DS5	YPM.12903	106601
C9	Agalma okenii	BWP 792-36	YPM.12903	106602
C10	Stephanophyes superba	BWP 571-9	YPM.12903	106603
C11	Marrus claudanielis	D1019-D9	YPM.12738	106604
C13	Erenna richardi	JSL II 1456 DS1	YPM.12903	106605
D5	Praya reticulata	KOK17_D Trawl022117	YPM.12903	106606
D6	Forskalia edwardsii	BWP 599-21 For13	YPM.12903	106607
D7	Lychnagalma utricularia	D331-D8	YPM.12903	106608
D8	Apolemia rubriversa	D651-D5	YPM.12903	106609
D9	Agalma okenii	D724-BW3-3	YPM.12903	106610
D11	Chelophyes appendiculata	D1019-BW01	YPM.12738	106611
D13	Halistemma rubrum	D335-D5	YPM.12903	106612
E1	Vogtia serrata	D856-SS3	YPM.12903	106613
E2	Bargmannia amoena	D861-D12	YPM.12903	106614
E3	Marrus claudanielis	D153-D7	YPM.12903	106615
E6	Frillagalma vityazi	D964-D8	YPM.12903	106616
E7	Lychnagalma utricularia	D962-D4	YPM.12903	106617
E9	Bargmannia amoena	JSL II 992-CGP3	YPM.12903	106618
E10	Desmophyes haematogaster	T847-D5	YPM.12903	106619
E11	Erenna laciniata	D1024-D4	YPM.12738	106620
F1	Bargmannia amoena	D861-D12	YPM.12903	106621
F2	Marrus orthocanna	D858-D11	YPM.12903	106622
F3	Lychnagalma utricularia	JSL II 1673-6	YPM.12903	106623
F4	Apolemia uvaria	NorthBergen 12-01-97	YPM.12903	106624
F6	Frillagalma vityazi	D665-D4	YPM.12903	106625
F7	Hippopodius hippopus	Discovery St. 7824#56	YPM.12903	106626
F9	Bargmannia amoena	JSL II 995-D3	YPM.12903	106627
F10	Cordagalma ordinatum	GOC D728-BW7-3	YPM.12903	106628
G3	Kephyes ovata	D856-D8	YPM.12903	106629
G4	Erenna laciniata	JSL II 1688-SS3	YPM.12903	106630
G6	Frillagalma vityazi	D457-SS3	YPM.12903	106631
G7A-B	Hippopodius hippopus	BWP 1027-12	YPM.12903	106632
G8	Cordagalma ordinatum	Oceanus 368-25-19A BWP	YPM.12903	106633
G10	Desmophyes haematogaster	T854-D5	YPM.12903	106634
G11	Forskalia asymmetrica	JSL II 987-D7	YPM.12903	106635
H1	Frillagalma vityazi	D858-SS4	YPM.12903	106636
H3	Bargmannia elongata	D494-SS9	YPM.12903	106637
H4	Cardianecta parchelion	JSL I 2633-DS1	YPM.12903	106638
H6	Agalma elegans	VF2016-5	YPM.12903	106639
H7A-B	Hippopodius hippopus	VF#7	YPM.12903	106640
H9	Agalma okenii	D723-BW3-1	YPM.12903	106641
H10	Apolemia rubriversa	D551-D5	YPM.12903	106642
H11	Sulculeolaria quadrivalvis	BWP 1051-3	YPM.12903	106643
I1	Vogtia serrata	D858-SS10	YPM.12903	106644
I3	Halistemma cupulifera	Discovery St. 3185	YPM.12903	106645
I6	Agalma elegans	SL0210	YPM.12903	106646
I7	Physalia physalis	TX026	YPM.12903	106647
I8	Cordagalma ordinatum	VFSM - Totton spec. C00	YPM.12903	106648
I9	Agalma elegans	RI3-7-18	YPM.12903	106649
I10	Gymnopraia lapislazula	V4098-D4	YPM.12940	106650
I11	Halistemma rubrum	JSL II 2656-DS6	YPM.12903	106651
J1	Nanomia bijuga	D860-BW1-C	YPM.12903	106652
J2	Desmophyes haematogaster	V2716-SS2	YPM.12903	106653
J4	Craseoa lathetica	D497-SS6	YPM.12903	106654
J6	Agalma elegans	BWD100212MB	YPM.12903	106655
J8A-B	Bargmannia elongata	D328_SS12	YPM.12903	106656
J9	Nanomia bijuga	D663-SS8	YPM.12903	106657
J10	Lilyopsis fluoracantha	V4098-SS4	YPM.12940	106658
J11	Stephanomia amphytridis	JSL I 2667-DS8	YPM.12903	106659
K2	Lychnagalma utricularia	JSL II 981-CGP6	YPM.12903	106660
K3	Sphaeronectes koellikeri	07-11-13 BW1	YPM.12903	106661
K6	Forskalia asymmetrica	JSL I 1407-D8	YPM.12903	106662
K7	Physalia physalis	Pp20130706WH	YPM.12903	106663
K8	Bargmannia elongata	D856-D3	YPM.12903	106664
K9	Diphyes dispar	BW1-2 17Sept2014	YPM.12903	106665
K10	Sphaeronectes koellikeri	V4098-SS5	YPM.12903	106666
K11	Cordagalma ordinatum	BWP 816-6	YPM.12903	106667
L1	Physophora hydrostatica	ENS43	YPM.12903	106668
L2	Lilyopsis medusa	WF BWP 30-09-06	YPM.12903	106669
L3	Forskalia tholoides	BWP 1072-2	YPM.12903	106670
L4	Rhizophysa eysenhardtii	BWP 862-3	YPM.12903	106671
L5	Erenna sirena	D860-D6	YPM.12903	106672
L6	Forskalia asymmetrica	JSL II 1684-D1	YPM.12903	106673
L7	Chelophyes appendiculata	BWP 24052004.00	YPM.12903	106674
L8	Bargmannia elongata	D960-SS3	YPM.12903	106675
L9	Forskalia edwardsii	D722-BW1-1	YPM.12903	106676
L10	Halistemma rubrum	VF 16-04-2003	YPM.12903	106677
L11	Erenna laciniata	D325-SS1	YPM.12903	106678
M2	Abylopsis tetragona	VFSS 13-04-11	YPM.12903	106679
M3	Apolemia lanosa	D858-D6	YPM.12903	106680
M7	Chelophyes appendiculata	VF2016-82	YPM.12903	106681
M8A-B	Stephanomia amphytridis	JSL I 3721-DS1	YPM.12903	106682
M9	Rhizophysa filiformis	BWP 567-8	YPM.12903	106683
M10	Halistemma rubrum	D720-D1	YPM.12903	106684
M11	Nanomia cara	BWP 1448-3	YPM.12903	106685
N1	Vogtia glabra	JSL II 959-D3	YPM.12903	106686
N2	Vogtia spinosa	Discovery St. 6662#2	YPM.12903	106687
N3	Halistemma transliratum	SL0301T	YPM.12903	106688
N6	Athorybia rosacea	D725-BW4-7	YPM.12903	106689
N7	Chelophyes appendiculata	VF#4	YPM.12903	106690
N8	Stephanomia amphytridis	JSL I 2688-CG6	YPM.12903	106691
N9	Physophora hydrostatica	D551-D11	YPM.12903	106692
N11	Sphaeronectes koellikeri	Manko_VIF18.95	YPM.12938	106693
O1	Agalma okenii	BWP 565-5	YPM.12903	106694
O4	Cordagalma ordinatum	BWP 370-6-6	YPM.12903	106695
O6	Athorybia rosacea	BWP 1035-18	YPM.12903	106696
O7	Apolemia lanosa	D614-D2	YPM.12903	106697
O8	Stephanomia amphytridis	D555-D7	YPM.12903	106698
O9	Physophora hydrostatica	EN543-T1	YPM.12903	106699
O10	Apolemia rubriversa	T673-D2	YPM.12903	106700
O11	Erenna laciniata	V2570-DS2	YPM.12903	106701
P1	Nanomia cara	1445-4	YPM.12903	106702
P2	Amphicaryon earnesti	Discovery St. 7856#24	YPM.12903	106703
P4	Nanomia cara	2138-S9	YPM.12903	106704
P6	Athorybia rosacea	BWP ATR-1010	YPM.12903	106705
P7	Apolemia lanosa	D668-SS1	YPM.12903	106706
P8	Sulculeolaria quadrivalvis	BWP 556-2	YPM.12903	106707
P9	Frillagalma vityazi	D556-D3	YPM.12903	106708
P10	Forskalia asymmetrica	JSL II 1680-D4	YPM.12903	106709
Q1	Rhizophysa eysenhardtii	SL0200ST	YPM.12903	106710
Q2	Agalma elegans	SL0205T	YPM.12903	106711
Q3	Apolemia rubriversa	D668-D7	YPM.12903	106712
Q4	Physonect sp	D666-SS11	YPM.12903	106713
Q6	Rosacea cymbiformis	D723-BW2-2/3	YPM.12903	106714
Q7	Apolemia lanosa	D552-SS8	YPM.12903	106715
Q8	Sulculeolaria quadrivalvis	BWP 512 SUQ 16	YPM.12903	106716
Q9	Athorybia rosacea	BWP 451.00	YPM.12903	106717
Q10	Erenna richardi	V2243-D? 1003m	YPM.12903	106718
Q11	Marrus claudanielis	T1037-D1	YPM.12903	106719
R1	Rhizophysa filiformis	BWP 566-17	YPM.12903	106720
R2	Rosacea cymbiformis	BWP 506.00	YPM.12903	106721
R3	Forskalia formosa	D666-D11	YPM.12903	106722
R4	Cordagalma bimaculatum	T1043-D1	YPM.12903	106723
R6	Rosacea cymbiformis	Voucher#196 BW4	YPM.12903	106724
R7	Vogtia serrata	D153-D6	YPM.12903	106725
R8	Sulculeolaria quadrivalvis	BWP 1061-17	YPM.12903	106726
R9	Abylopsis tetragona	VF#15	YPM.12903	106727
R10	Resomia ornicephala	D1025-D10	YPM.12738	106728
R11	Erenna richardi	JSL II 1456-D1	YPM.12903	106729
S2	Abyla bicarinata	BWP 2053-13	YPM.12903	106730
S3	Chelophyes appendiculata	V14	YPM.12903	106731
S5	Resomia ornicephala	D965-D7	YPM.12903	106732
S7	Vogtia serrata	10Dec09-Trawl	YPM.12903	106733
S8	Praya dubia	JSL II 1684-2 (9)	YPM.12903	106734
S10	Resomia ornicephala	D1025-SS5	YPM.12738	106735
S11	Erenna richardi	T751-DS03	YPM.12903	106736
T2	Athorybia rosacea	BWP 1036-10	YPM.12903	106737
T3	Halistemma rubrum	D339-D12	YPM.12903	106738
T4	Lensia conoidea	D500-D4	YPM.12903	106739
T6	Abylopsis tetragona	SL0303S	YPM.12903	106740
T7	Vogtia serrata	D856-SS9	YPM.12903	106741
T8	Praya dubia	D962-T1	YPM.12903	106742
T9	Craseoa lathetica	D330-S11	YPM.12903	106743
T10	Resomia ornicephala	D1025-SS6	YPM.12738	106744
T11	Praya dubia	JSL II 990-CGP2	YPM.12903	106745
U1	Forskalia asymmetrica	T595-SS3	YPM.12903	106746
U2	Halistemma foliacea	SL0401ST	YPM.12903	106747
U3	Craseoa lathetica	D497-SS6	YPM.12903	106748
U5	Resomia dunni	D963-D4	YPM.12903	106749
U6	Abylopsis tetragona	JSL I 2950-CG-7	YPM.12903	106750
U7	Craseoa lathetica	T1043-SS7	YPM.12903	106751
U8	Bargmannia amoena	JSL I 2657-D8	YPM.12903	106752
U9	Hippopodius hippopus	VF#8	YPM.12903	106753
U10	Gymnopraia lapislazula	D1020-SS1	YPM.12738	106754
U11	Praya dubia	JSL II 1678-DS7	YPM.12903	106755
V1	Forskalia edwardsii	BWP 1042-6	YPM.12903	106756
V2	Physalia physalis	WH 20130706 Corm#3	YPM.12903	106757
V3	Rosacea plicata	WF Trawl 31-07-07	YPM.12903	106758
V4	Agalma clausi	BWP 1092-6	YPM.12903	106759
V5	Lilyopsis fluoracantha	D963-D8	YPM.12903	106760
V6	Abylopsis tetragona	JSL I 2953-CG-3	YPM.12903	106761
V7	Craseoa lathetica	D614-D4	YPM.12903	106762
V8	Desmophyes haematogaster	V3642-SS3	YPM.12903	106763
V10	Gymnopraia lapislazula	D1022-SS3	YPM.12738	106764
V11	Desmophyes haematogaster	T749-SS6	YPM.12903	106765
W0	Erenna sirena	D860-D6	YPM.12903	106766
W1	Ceratocymba leuckarti	BWP 1034-4	YPM.12903	106767
W2	Erenna richardi	JSL II D1456-DS1	YPM.12903	106768
W3	Bassia bassensis	BWP 1497-15+16	YPM.12903	106769
W4	Bargmannia elongata	D554-SS3	YPM.12903	106770
W5	Gymnopraia lapislazula	D965-D4	YPM.12903	106771
W6	Diphyes dispar	BWP 567-16	YPM.12903	106772
W7	Craseoa lathetica	D611-SS7	YPM.12903	106773
W8	Chuniphyes multidentata	D493-SS2	YPM.12903	106774
W9	Chuniphyes multidentata	D107-S9	YPM.12903	106775
W10	Gymnopraia lapislazula	D1022-SS5	YPM.12738	106776
X1	Resomia persica	D343-D9	YPM.12903	106777
X2	Sulculeolaria quadrivalvis	1349-22	YPM.12903	106778
X4	Physophora hydrostatica	EN182 1845-5	YPM.12903	106779
X5	Chuniphyes moserae	D959-SS3	YPM.12903	106780
X6	Diphyes dispar	D667-BW4-3	YPM.12903	106781
X7	Rhizophysa eysenhardtii	BWP 634-6	YPM.12903	106782
X8	Sphaeronectes koellikeri	BWP 657-8	YPM.12903	106783
X9	Physalia physalis	YPM IZ 035012	TBD	
X10	Chuniphyes multidentata	D1023-SS12	YPM.12738	106784
X11	Resomia ornicephala	T1157-SS10	YPM.12903	106785
Y1	Diphyes bojani	BWP 1060-2	YPM.12903	106786
Y2	Bargmannia elongata	D153-SS1	YPM.12903	106787
Y4	Nectadamas richardi	Discovery St. 10111#6	YPM.12903	106788
Y5	Nanomia bijuga	BWP 1048-17	YPM.12903	106789
Y6	Diphyes dispar	BWP 1503-2	YPM.12903	106790
Y7	Rhizophysa eysenhardtii	BWP 794-3	YPM.12903	106791
Y8	Marrus claudanielis	D959-SS2	YPM.12903	106792
Y10	Chuniphyes multidentata	D1025-SS9	YPM.12738	106793
Y11	Resomia ornicephala	T847-SS11	YPM.12903	106794
Z1	Ceratocymba dentata	BWP 933-2	YPM.12903	106795
Z2	Stephanophyes superba	1345-14	YPM.12903	106796
Z5	Nanomia bijuga	21-Sep-14	YPM.12903	106797
Z6	Rhizophysa filiformis	BWP 1565-4	YPM.12903	106798
Z7	Rhizophysa eysenhardtii	BWP 814-3	YPM.12903	106799
Z8	Marrus claudanielis	T1105-D5	YPM.12903	106800
Z9	Nanomia cara	BWP 1420-10	YPM.12903	106801
Z11	Sphaeronectes koellikeri	BWP 528005.00	YPM.12903	106802
