# Supplementary material for: The Evolutionary History of Siphonophore Tentilla: Novelties, Convergence, and Integration
Source: Integr Org Biol. 2021 May 26;3(1):obab019. doi: 10.1093/iob/obab019 (PMC8331849; doi:10.1093/iob/obab019)
Supplement: obab019_Supplementary_Data [file obab019_supplementary_data.zip › SM17-species_data.pdf]

SM17-species_data

Species	N	Heteroneme free length µm	Heteroneme width µm	Heteroneme volume µm3	Heteroneme shaft free length µm	Heteroneme shaft width µm	Heteroneme number	Haploneme free length µm	Haploneme width µm	Desmoneme length µm	Desmoneme width µm
Abyla bicarinata	1	111.20	7.80	3542.37	76.50		8.00	59.20	9.30	18.60	11.80
Abylopsis tetragona	5	132.90±10.96	13.82±1.62	14487.09±3868.23	84.44±13.28	5.30±0.62	9.60±0.98	41.74±4.17	8.42±0.90	19.40±2.10	12.92±1.87
Agalma clausi	1	80.00	14.90	9299.55	52.30	10.40	8.00	45.60	6.20	8.60	5.30
Agalma elegans	5	107.72±6.84	16.66±0.69	15967.91±2041.91	76.46±4.65	5.96±0.55	10.40±1.50	31.90±1.91	4.80±0.27	5.42±0.22	4.50±0.24
Agalma okenii	5	114.40±10.44	20.24±1.06	25067.26±3755.95	62.10±7.26	6.24±0.98	49.00±5.17	53.80±2.73	9.32±0.86	6.70±0.40	4.82±0.47
Amphicaryon earnesti	1	18.90	9.60	912.02			4.00	17.50	4.00	5.40	5.10
Apolemia lanosa	5	22.41±1.45	14.47±0.46	2493.14±289.19	35.88±7.39	3.65±0.46					
Apolemia rubriversa	5	17.56±3.92	10.24±1.57	1378.98±838.95	24.22±3.24	2.48±0.43					
Apolemia uvaria	1	20.90	12.80	1792.94	36.60	3.20					
Athorybia rosacea	5	40.40±3.48	15.00±0.92	5002.25±909.85	35.28±3.49	4.90±0.97	11.60±3.25	24.88±1.45	4.50±0.08	7.92±1.05	6.32±1.23
Bargmannia amoena	5	88.16±4.13	62.66±2.72	185304.98±23180.03	62.96±5.73	13.24±0.85	8.40±1.81	14.28±2.60	12.02±1.67		
Bargmannia elongata	6	89.34±3.42	60.38±2.96	175290.02±25580.05	62.52±5.55	9.82±0.83	3.60±0.37	8.20±0.83	6.88±0.70		
Bargmannia lata	1	158.60	114.10	1081120.52	135.60	19.10	12.00	17.80	13.70		
Bassia bassensis	1	73.80	10.30	4099.50	49.80	2.90	8.00	41.60	6.80	19.90	10.00
Bathyphysa conifera	1						0.00	21.80	20.80		
Cardianecta parchelion	1	42.00	13.20	3831.75	16.70	4.50		34.90	4.80	15.30	12.40
Ceratocymba dentata	1	154.30	13.20	14077.11	138.00	3.60	18.00	37.40	7.10	12.90	6.80
Ceratocymba leuckarti	1	108.00	9.30	4890.91	94.10	2.70	8.00	45.40	8.20	17.40	8.70
Chelophyes appendiculata	5	59.84±2.30	5.56±0.20	978.46±88.92	41.58±2.82	2.14±0.24	7.60±0.75	22.16±1.78	3.96±0.28	11.04±1.87	6.52±0.92
Chuniphyes moserae	1	66.00	7.80	2102.48	156.40	9.10	12.00	35.70	5.70	9.40	7.20
Chuniphyes multidentata	5	96.36±7.71	7.56±0.46	2994.28±516.01	79.58±7.76	2.82±0.31	26.40±5.34	35.56±1.58	4.66±0.27	11.82±1.01	7.88±0.53
Cordagalma bimaculatum	1	27.40	13.40	2576.08	20.00	3.00	5.00	20.70	4.90	11.10	6.30
Cordagalma ordinatum	5	14.08±0.64	6.00±0.42	276.24±42.91	7.08±0.37	1.56±0.16	5.00	17.48±1.79	1.95±0.20		
Craseoa lathetica	6	91.98±9.63	8.12±0.20	3137.79±267.96	67.37±4.67	3.37±0.32	34.67±0.84	45.07±2.69	6.12±0.43	14.33±1.85	9.77±1.33
Desmophyes haematogaster	5	81.60±23.99	10.54±0.67	4324.38±762.45	63.04±17.33	3.64±0.34	20.80±10.07	33.22±5.09	4.90±0.68	12.64±1.46	8.02±1.18
Diphyes bojani	1	60.80	5.40	928.31	36.70	2.10	6.00	21.70	3.00	8.60	5.10
Diphyes dispar	5	91.16±8.15	9.16±0.79	4255.62±956.80	67.88±9.01	2.74±0.11	8.00±0.89	30.50±2.72	5.18±0.47	14.22±1.02	8.10±0.60
Erenna laciniata	4	126.95±13.66	19.45±1.09	25872.74±4679.48	102.90±12.58	5.63±0.77	251.50±57.53	34.43±6.74	14.73±1.95		
Erenna richardi	5	163.52±10.26	28.52±0.86	70727.49±8097.84	126.62±4.96	9.44±0.87	1290.40±190.31	40.12±6.67	16.84±1.82		
Erenna sirena	2	158.70±23.20	18.55±0.05	28616.05±4334.16	131.05±21.25	5.75±1.75	237.00±13.00	29.70±6.10	20.85±0.65		
Forskalia asymmetrica	5	48.40±4.80	17.42±0.81	7858.57±1271.36	33.20±3.96	4.60±0.40	116.00±20.07	41.18±7.05	7.04±0.43	13.66±1.11	8.50±0.38
Forskalia edwardsii	5	33.90±1.34	13.82±0.52	3445.58±377.99	24.38±2.33	3.24±0.25	32.00±2.76	30.70±2.34	6.02±0.41	10.70±1.05	7.00±0.81
Forskalia formosa	1	37.80	16.90	5652.82	23.10	4.60	60.00	39.70	6.80	14.50	10.50
Forskalia tholoides	1	43.60	21.80	10849.23	28.30	5.80		40.60	6.30	14.20	8.00
Frillagalma vityazi	5	82.00±4.81	24.52±4.28	29789.16±10375.37	60.00±5.31	6.76±1.28	3.20±0.20	46.18±4.07	6.32±0.69		
Gymnopraia lapislazula	5	87.50±2.19	10.58±0.31	5166.92±381.99	65.52±4.77	3.06±0.35	2.00	27.12±1.61	5.32±0.47	10.12±0.41	6.56±0.53
Halistemma cupulifera	1	43.50	22.50	11530.65	31.30	7.20				30.70	19.80
Halistemma foliacea	1	83.80	31.10	42438.93	53.50	9.20		63.50	11.90	31.60	17.10
Halistemma rubrum	5	52.32±5.85	16.50±1.86	8468.34±2280.70	33.92±4.25	4.52±0.39	134.00±7.64	43.40±5.22	6.78±0.54	12.16±2.75	6.52±1.16
Halistemma transliratum	1	72.80	25.40	24592.26	58.60	8.50		53.30	12.10	19.30	9.10
Hippopodius hippopus	5	63.78±2.85	9.44±0.85	3110.91±640.38	46.74±3.18	2.78±0.17	8.40±0.40	25.58±1.98	4.26±0.25	11.14±1.45	6.88±0.82
Kephyes ovata	1	85.10	6.70	2000.22	73.90	2.00	12.00	32.90	4.20	12.60	7.90
Lensia conoidea	5	58.82±5.07	6.46±0.30	1319.60±205.87	43.38±5.02	2.16±0.27	6.80±0.49	23.92±1.51	4.18±0.26	9.54±0.98	5.96±0.87
Lilyopsis fluoracantha	3	157.80±7.77	16.27±1.74	22713.28±5164.46	134.20±13.70	5.30±0.55	28.00±4.62	61.50±3.04	9.17±0.62	20.67±2.43	10.97±0.32
Lilyopsis medusa	1	40.50	4.30	392.10	25.60	2.80	20.00	26.50	4.20	22.80	13.20
Lychnagalma utricularia	5	79.42±2.05	18.78±1.15	14833.25±1767.39	56.84±2.65	6.20±0.39	580.40±72.75	50.84±4.56	8.84±1.20		
Marrus claudanielis	5	88.20±4.04	22.66±0.61	23809.12±1737.24	60.36±9.02	5.28±0.31	276.40±51.48	44.24±3.73	6.84±0.82	16.04±1.73	10.40±1.20
Marrus orthocanna	1	49.10	12.00	3702.06	40.30	5.80	38.00	31.30	4.00	14.30	8.40
Nanomia sp	11	31.59±2.21	15.16±1.31	4537.73±1154.50	22.40±1.76	4.45±0.47	128.36±51.05	29.99±2.92	5.93±0.56	14.42±1.29	7.14±0.51
Nectadamas richardi	1	73.80	14.60	8236.86			6.00	74.40	9.00		
Physalia physalis	5						0.00	23.54±1.81	20.96±1.65		
Physonect sp	1	126.30	19.40	24888.94	80.70	6.30	54.00	75.20	9.20	19.50	13.90
Physophora gilmeri	1	102.00	26.30	36941.23	70.00	7.00	104.00	53.40	9.00		
Physophora hydrostatica	5	80.86±9.55	19.08±2.53	18279.72±5987.28	59.34±6.13	5.90±0.43	33.60±8.38	40.66±5.08	7.12±0.97	7.03±0.37	5.23±0.29
Praya dubia	5	140.20±12.06	13.56±1.06	13593.61±1962.19	103.40±15.56	4.90±0.46	36.00±4.60	69.32±7.20	8.04±0.37	13.88±1.24	9.26±1.04
Praya reticulata	1	207.90	15.00	24492.70	167.70	7.90	72.00	97.90	10.90	18.50	13.00
Resomia dunni	1	277.10	44.80	291200.55	233.30	15.40		93.90	11.50	36.70	18.10
Resomia ornicephala	6	67.37±1.72	17.05±1.19	10614.00±1720.95	49.98±2.74	5.58±0.21	182.83±18.01	63.65±2.98	9.33±0.48	19.25±0.46	16.13±0.75
Resomia persica	1	172.00	37.40	125971.21	135.30	7.90		87.20	12.40	30.60	16.20
Rhizophysa eysenhardtii	5						0.00	16.90±1.12	14.26±1.20		
Rhizophysa filiformis	5						0.00	24.82±2.05	22.86±1.82		
Rosacea cymbiformis	3	72.93±16.37	9.20±2.47	4570.63±3191.27	56.03±7.81	3.53±1.60	12.67±2.91	35.03±2.84	6.07±1.32	13.30±2.03	8.77±0.38
Rosacea plicata	1	89.50	10.40	5068.62	69.10	4.70	24.00	33.20	4.50	12.30	6.70
Sphaeronectes koellikeri	5	24.88±1.46	4.98±0.10	321.11±10.74	16.44±2.00	1.62±0.17	4.80±0.49	11.92±0.49	2.00±0.09	6.18±0.58	3.34±0.29
Stephanomia amphytridis	5	161.98±3.46	43.50±1.10	160586.10±7204.17	125.00±2.33	14.10±0.38	425.50±52.90	130.68±5.69	18.42±0.77	42.12±3.98	26.06±2.99
Stephanophyes superba	2	96.35±6.05	8.58±1.48	3899.43±1515.99	72.30±8.50	3.83±0.03	35.00±1.00	42.40±0.80	6.80±0.50	16.88±3.98	11.55±0.95
Sulculeolaria quadrivalvis	5	31.80±2.81	4.80±0.49	425.31±119.70	23.30±2.15	1.82±0.19	7.60±0.40	18.60±2.22	3.48±0.34	9.48±1.78	5.52±1.17
Thermopalia taraxaca	1	33.80	14.00	3468.75	23.30	3.00				11.90	5.00
Vogtia glabra	1	115.80	24.80	37291.65	73.20	7.80	8.00	34.50	6.60	13.70	6.60
Vogtia serrata	5	163.96±7.43	23.56±0.38	47839.47±3141.75	109.56±5.36	6.40±0.68	12.80±1.50	53.32±1.93	7.00±0.31	15.36±0.99	9.12±0.67
Vogtia spinosa	1	144.20	27.30	56271.72	93.40	11.20	6.00	39.80	5.70	16.10	9.10

Species	Rhopaloneme length µm	Rhopaloneme width µm	Cnidoband length µm	Cnidoband free length µm	Cnidoband width µm	Haploneme row number	Tentacle width µm	Pedicle width µm	Elastic strand width µm	Involucrum length µm	Cnidoband coiledness
Abyla bicarinata	12.70	3.20	308.30	308.30	50.90	7		16.30	12.20	0.00	1.00
Abylopsis tetragona	15.08±1.97	3.98±0.57	578.84±83.77	619.00±89.97	58.18±3.33	7	43.68±7.29	24.40±3.26	13.92±3.04	75.74±21.05	1.07±0.01
Agalma clausi	7.20	1.90	274.80	850.50	123.60	18	175.20	43.40	24.40	662.60	3.09
Agalma elegans	7.62±0.43	2.00±0.11	342.88±46.62	1338.74±131.87	66.68±6.04	13±0.77	90.10±6.71	60.66±6.87	22.68±2.67	336.44±19.17	3.98±0.18
Agalma okenii	8.56±1.13	2.24±0.39	789.90±87.84	4333.94±387.63	107.26±8.19	13.60±0.40	112.58±10.29	53.24±7.65	24.74±1.35	866.06±98.37	5.63±0.59
Amphicaryon earnesti	9.90	2.20	57.40	62.20	26.30	7					1.08
Apolemia lanosa							51.30±5.24				
Apolemia rubriversa							28.66±10.06				
Apolemia uvaria							76.10				
Athorybia rosacea	7.50±0.38	2.18±0.14	188.72±36.34	385.18±82.55	54.00±2.43	10±1.18	92.68±9.92	53.12±12.87	9.70±0.99	154.68±16.67	2.01±0.25
Bargmannia amoena			2549.62±1020.73	3782.32±1698.80	95.94±17.52	6.40±1.60	201.52±35.61	60.80±7.29		0.00	1.34±0.10
Bargmannia elongata			1584.24±421.17	2232.76±611.33	40.50±5.27	5.60±0.89	199.28±28.02	60.34±14.83		0.00	1.43±0.09
Bargmannia lata			4297.10	12682.70	100.40	7		145.70		0.00	2.95
Bassia bassensis	10.00	3.40	224.80	237.40	53.30	7	37.10	26.20	8.60	26.10	1.06
Bathyphysa conifera							2000.00				
Cardianecta parchelion			537.60		41.30	9		91.70	33.80	727.20	
Ceratocymba dentata	10.80	3.40	416.60	416.60			44.80	43.10	6.60	0.00	1.00
Ceratocymba leuckarti	13.40	3.40	304.70	331.80	57.00	7	38.70	18.80	14.10	25.50	1.09
Chelophyes appendiculata	9.80±1.21	2.52±0.21	156.62±18.34	187.98±23.85	28.94±6.00	7	28.80±8.70	7.16±1.50	6.22±0.72	15.46±3.29	1.21±0.09
Chuniphyes moserae	9.30	2.20	158.90	232.50	40.30	7		43.50		0.00	1.46
Chuniphyes multidentata	11.70±1.10	2.72±0.15	222.64±21.14	317.14±35.66	36.98±4.47	7	48.88±9.23	20.26±1.07	9.38±1.58	18.70±11.97	1.43±0.07
Cordagalma bimaculatum			108.30	108.30	76.50	7	31.20	17.20		0.00	1.00
Cordagalma ordinatum			40.50±4.39	40.50±4.39	31.72±1.61	9.50±0.58	15.14±3.79	8.46±2.36	3.66±0.22	9.24±2.38	1.00
Craseoa lathetica	19.05±3.46	4.78±1.16	264.35±25.01	462.77±33.04	60.42±5.42	7	126.38±39.07	28.50±7.89	15.95±4.70	87.48±37.15	1.79±0.15
Desmophyes haematogaster	15.66±2.52	4.02±0.38	212.14±33.97	296.36±44.56	44.76±4.87	7	55.52±15.52	36.16±10.78	13.20±4.18	16.00±11.01	1.41±0.05
Diphyes bojani	7.30	1.60	132.70	157.50				13.60		22.80	1.19
Diphyes dispar	13.42±1.39	3.98±0.24	211.82±16.26	253.90±15.84	36.40±2.79	7	49.14±7.87	16.72±2.73	9.12±1.02	38.00±7.65	1.22±0.11
Erenna laciniata			2369.80±408.88	2669.10±301.78	238.83±71.08	19.75±4.03	1220.00±164.52	219.60±46.57		0.00	1.16±0.07
Erenna richardi			18108.20±2174.89	18208.40±2154.01	796.10±174.40	36.80±4.41	1853.78±345.97	465.66±65.77		0.00	1.01±0.01
Erenna sirena			4360.55±113.55	4360.55±113.55	352.60±88.40	16±4	1280.00	187.40±17.30		0.00	1.00
Forskalia asymmetrica	14.46±1.60	4.86±0.44	975.34±104.15	2767.06±170.52	156.10±20.44	19.60±1.17	197.28±44.58	82.30±11.04	24.16±3.14	0.00	2.93±0.26
Forskalia edwardsii	10.86±0.90	3.28±0.28	322.18±19.69	832.46±34.08	87.46±5.60	14.20±0.73	69.84±9.01	51.00±7.73	17.54±0.59	0.00	2.63±0.21
Forskalia formosa	13.00	5.70	751.90	1668.60	113.00	16	100.00	43.70	8.70	0.00	2.22
Forskalia tholoides	11.00	4.30	601.40	1378.00	101.50	16	106.50	62.50	11.20	0.00	2.29
Frillagalma vityazi			139.72±16.52	183.82±22.29	46.18±11.59	5	61.22±4.46	27.38±6.77	7.76±0.87	121.96±32.11	1.33±0.11
Gymnopraia lapislazula	11.10±0.49	2.80±0.23	182.36±13.93	224.10±10.93	41.92±1.71	7	74.04±13.67	21.18±4.45	14.86±1.78	30.36±12.01	1.25±0.06
Halistemma cupulifera	20.40	5.40	686.40	2496.00	97.20	21		58.50	23.80	37.20	3.64
Halistemma foliacea	28.10	11.30	3392.90	12904.80	421.70	36		264.20	182.30	487.20	3.80
Halistemma rubrum	19.20±5.14	4.78±0.55	1475.66±257.41	5269.90±1280.20	196.46±39.43	23.60±1.94	232.44±65.97	81.24±22.15	50.48±7.72	28.18±23.76	3.45±0.42
Halistemma transliratum	26.20	10.00	1673.60	7780.40	252.70	24		175.00		261.30	4.65
Hippopodius hippopus	11.64±1.08	2.48±0.20	106.00±4.42	162.60±15.48	42.86±9.70	7	59.46±13.05	12.44±1.62	7.02±0.71	43.30±10.12	1.55±0.18
Kephyes ovata	12.90	3.20	112.50	121.00	54.50	7	34.60	18.70	8.50	12.30	1.08
Lensia conoidea	10.80±0.75	2.54±0.14	148.32±4.33	186.82±11.33	35.12±1.85	6.60±0.40	23.48±4.64	14.10±2.33	8.14±0.64	22.42±4.56	1.26±0.07
Lilyopsis fluoracantha	24.57±0.71	6.27±0.62	597.00±38.93	837.53±72.27	102.47±9.02	7	219.57±140.22	23.43±1.20	16.63±2.61	82.07±37.69	1.43±0.20
Lilyopsis medusa	13.00	3.20	254.60	400.60	43.30	7	52.80	44.40	7.70	131.40	1.57
Lychnagalma utricularia			2253.50±242.45	22427.70±6709.99	199.54±7.17	19.80±1.98	599.30±97.74	166.00±30.02	79.98±5.96	2150.62±241.03	9.65±2.28
Marrus claudanielis	18.70±1.25	5.44±0.79	5990.86±3634.93	10092.48±2879.48	133.50±11.78	17.20±1.43	299.14±62.36	100.70±15.78	31.58±3.83	22.72±22.72	3.14±0.78
Marrus orthocanna	13.80	4.40	691.40	691.40	102.60	16	80.70	38.60	14.30	27.50	1.00
Nanomia sp	15.69±1.57	4.23±0.48	633.65±151.94	2810.52±744.37	106.27±15.75	17.73±1.74	120.47±25.81	56.72±13.27	40.84±14.40	238.72±79.15	4.21±0.19
Nectadamas richardi								9.20	6.70	0.00	
Physalia physalis			724.54±147.97	778.54±236.01	295.34±64.70	29.60±5.45	517.42±169.52	169.66±30.33		0.00	1.04±0.15
Physonect sp	31.90	12.50	774.60	783.50	230.50	26	156.80	52.70	32.40	0.00	1.01
Physophora gilmeri			989.00	4638.20	170.60	18	500.00	228.20	42.30	1606.10	4.69
Physophora hydrostatica	12.23±1.25	2.67±0.16	702.90±132.40	3523.74±732.75	85.78±13.61	13±1.48	218.02±28.08	98.86±26.36	23.40±3.60	791.22±144.07	4.92±0.17
Praya dubia	23.52±2.37	5.02±0.28	321.12±27.54	701.66±54.92	65.86±7.49	7	93.70±13.27	39.38±6.51	10.62±1.06	18.68±11.44	2.19±0.04
Praya reticulata	33.30	7.60	671.70	1913.90	78.10	7		99.40	24.40	0.00	2.85
Resomia dunni	28.90	10.70		12820.00	342.50	27		136.40	101.80		
Resomia ornicephala	24.95±0.40	6.52±0.30	1685.30±89.47	4984.73±334.54	155.92±21.26	23±0.58	216.92±37.42	133.92±15.16	51.43±11.30	419.43±77.30	3.29±0.10
Resomia persica	23.70	3.50	1458.60	7060.50	207.50	14	191.00	184.60	75.70	0.00	4.84
Rhizophysa eysenhardtii			1132.46±319.41	1132.46±319.41	50.58±13.73	2.60±0.60	175.34±49.11	38.40±8.87		0.00	1.00
Rhizophysa filiformis			153.10±18.65	153.10±18.65	112.98±6.27	6.80±0.58	262.82±31.61	87.38±11.52		0.00	1.00
Rosacea cymbiformis	12.47±0.91	3.27±0.48	235.93±104.89	336.03±78.19	41.97±9.56	7	50.60±11.82	27.63±15.33	7.77±2.92	68.83±18.45	1.70±0.35
Rosacea plicata	18.60	4.80	252.00	261.10	53.00	7	74.00	15.10	4.10	46.40	1.04
Sphaeronectes koellikeri	6.14±0.42	1.58±0.18	45.76±2.19	56.98±3.49	15.00±1.10	7	12.58±2.07	5.16±0.44	2.66±0.20	14.16±3.15	1.24±0.03
Stephanomia amphytridis	45.06±2.61	12.74±0.80	7952.28±739.24	21768.90±2225.54	734.91±71.02	43.40±5.56	1137.13±92.54	404.40±70.59	377.12±38.10	0.00	2.73±0.09
Stephanophyes superba	20.55±1.95	4.05±0.05	543.78±60.23	978.00±120.00	70.98±10.98	7	64.38±19.78	27.95±5.95	11.85±2.05	56.75±12.75	1.80±0.02
Sulculeolaria quadrivalvis	8.14±1.34	2.14±0.38	90.04±5.31	122.86±13.16	26.64±4.56	7	26.78±3.37	10.86±1.88	6.08±0.89	17.52±3.38	1.36±0.11
Thermopalia taraxaca	18.80	4.30									
Vogtia glabra	13.50	2.80	318.40	599.10	54.10	7	51.40	8.50	8.90	76.00	1.88
Vogtia serrata	17.74±0.62	4.70±0.43	616.30±82.94	1361.64±232.43	71.70±3.36	7	199.52±25.47	48.88±9.75	16.04±3.03	153.40±48.24	2.19±0.17
Vogtia spinosa	10.60	3.00	407.40	1007.60	50.00	7	97.80	16.60	10.10	88.70	2.47

Species	Heteroneme elongation	Haploneme elongation	Desmoneme elongation	Rhopaloneme elongation	Heteroneme shaft extension	Heteroneme/Cnidoband length	Total heteroneme volume µm3	Total haploneme volume µm3	log Total nematocyst volume	Haploneme surface area:volume
Abyla bicarinata	14.26	6.37	1.58	3.97	1.45	0.36	28338.94	88874.42	11.67	0.50
Abylopsis tetragona	10.09±1.27	5.05±0.41	1.60±0.20	3.89±0.31	1.86±0.52	0.23±0.03	143262.09±48176.59	130946.61±42940.59	12.31±0.33	0.59±0.07
Agalma clausi	5.37	7.35	1.62	3.79	1.53	0.09	74396.44	125901.08	12.21	0.76
Agalma elegans	6.47±0.32	6.68±0.36	1.21±0.06	3.82±0.13	1.43±0.12	0.08±0.01	165284.89±29261.67	104900.73±6708.06	12.47±0.13	0.99±0.05
Agalma okenii	5.67±0.49	5.96±0.64	1.41±0.06	4.18±0.77	1.89±0.13	0.03±0.00	1211423.09±208348.04	1154217.39±179183.71	14.63±0.15	0.52±0.04
Amphicaryon earnesti	1.97	4.38	1.06	4.50		0.30	3648.08	2279.75	8.69	1.19
Apolemia lanosa	1.55±0.08				0.82±0.26	22405.00±1454.14			7.79±0.12	
Apolemia rubriversa	1.66±0.09				0.75±0.14	17560.00±3919.90			6.72±0.44	
Apolemia uvaria	1.63				0.57	20900.00			7.49	
Athorybia rosacea	2.68±0.12	5.52±0.25	1.31±0.09	3.49±0.24	1.16±0.08	0.12±0.02	68786.90±23946.59	22879.57±5287.26	11.09±0.46	1.05±0.01
Bargmannia amoena	1.41±0.02	1.18±0.12			1.44±0.11	0.06±0.02	1648782.82±515923.18	525559.68±369599.94	14.30±0.37	0.51±0.06
Bargmannia elongata	1.48±0.04	1.20±0.07			1.47±0.10	0.07±0.02	643747.91±127495.89	74646.42±31768.31	13.37±0.22	0.86±0.08
Bargmannia lata	1.39	1.30			1.17	0.01	12973446.22	1619387.98	16.50	0.40
Bassia bassensis	7.17	6.12	1.99	2.94	1.48	0.31	32795.97	35162.65	11.13	0.69
Bathyphysa conifera		1.05								0.28
Cardianecta parchelion	3.18	7.27	1.23		2.51				8.25	0.98
Ceratocymba dentata	11.69	5.27	1.90	3.18	1.12	0.37	253387.93	57922.57	12.65	0.67
Ceratocymba leuckarti	11.61	5.54	2.00	3.94	1.15	0.33	39127.25	64676.23	11.55	0.57
Chelophyes appendiculata	10.79±0.42	5.73±0.71	1.70±0.19	3.86±0.17	1.47±0.13	0.33±0.03	7441.08±1042.05	9096.09±2257.74	9.66±0.16	1.22±0.09
Chuniphyes moserae	8.46	6.26	1.31	4.23	0.42	0.28	25229.81	24772.20	10.82	0.83
Chuniphyes multidentata	12.79±0.84	7.66±0.17	1.50±0.08	4.28±0.22	1.23±0.06	0.32±0.04	80685.78±22601.70	28468.32±4868.20	11.45±0.28	1.02±0.06
Cordagalma bimaculatum	2.04	4.22	1.76		1.37	0.25	12880.41	5751.66	9.83	0.97
Cordagalma ordinatum	2.37±0.10	9.07±0.88			1.94±0.11	0.36±0.03	1381.19±214.54	676.73±140.66	7.23±0.35	2.49±0.22
Craseoa lathetica	11.44±1.36	7.46±0.42	1.48±0.09	4.44±0.54	1.37±0.11	0.20±0.03	108050.81±7543.86	69673.77±13069.82	12.07±0.09	0.79±0.05
Desmophyes haematogaster	8.33±2.99	6.79±0.44	1.65±0.18	3.92±0.53	1.30±0.10	0.27±0.05	87505.20±37036.55	31272.71±12259.55	11.31±0.43	1.03±0.13
Diphyes bojani	11.26	7.23	1.69	4.56	1.66	0.39	5569.83	5368.59	9.30	1.57
Diphyes dispar	10.16±0.96	6.23±1.07	1.77±0.10	3.36±0.28	1.39±0.15	0.36±0.04	32613.37±6074.53	21259.31±3736.05	10.83±0.18	0.95±0.10
Erenna laciniata	6.52±0.59	2.31±0.31			1.25±0.10	0.05±0.00	6845105.30±2238084.30	747922.59±184631.62	15.68±0.35	0.36±0.05
Erenna richardi	5.72±0.24	2.39±0.27			1.30±0.08	0.01±0.00	89002810.23±14106736.14	6373424.78±1334039.77	18.34±0.13	0.31±0.03
Erenna sirena	8.55±1.23	1.43±0.34			1.21±0.02	0.04±0.00	6838347.69±1399205.55	1398380.19±209728.06	15.91±0.15	0.26±0.00
Forskalia asymmetrica	2.78±0.24	5.86±1.10	1.61±0.13	2.95±0.07	1.47±0.05	0.02±0.00	831183.20±120689.03	437509.28±102009.19	12.99±1.05	0.66±0.03
Forskalia edwardsii	2.45±0.04	5.23±0.63	1.54±0.04	3.39±0.38	1.43±0.12	0.04±0.00	108875.00±11476.85	79783.81±6853.86	12.14±0.07	0.80±0.05
Forskalia formosa	2.24	5.84	1.38	2.28	1.64	0.02	339168.96	235857.82	13.26	0.69
Forskalia tholoides	2.00	6.44	1.78	2.56	1.54	0.03		184550.16	12.13	0.75
Frillagalma vityazi	3.73±0.64	7.44±0.40			1.39±0.08	0.47±0.04	93914.35±30682.13	31173.07±7984.35	11.50±0.36	0.79±0.10
Gymnopraia lapislazula	8.28±0.17	5.16±0.19	1.58±0.13	4.04±0.28	1.37±0.11	0.39±0.02	10333.83±763.99	17635.09±3015.26	10.20±0.14	0.92±0.08
Halistemma cupulifera	1.93		1.55	3.78	1.39	0.02			9.35	
Halistemma foliacea	2.69	5.34	1.85	2.49	1.57	0.01		5105879.81	15.45	0.39
Halistemma rubrum	3.22±0.22	6.36±0.40	1.84±0.11	3.90±0.71	1.56±0.08	0.01±0.00	1556279.37±116648.17	990123.19±396535.32	13.90±0.62	0.71±0.06
Halistemma transliratum	2.87	4.40	2.12	2.62	1.24	0.01		2627320.94	14.78	0.39
Hippopodius hippopus	6.96±0.64	6.00±0.28	1.61±0.04	4.67±0.10	1.38±0.07	0.41±0.04	26043.91±5154.41	9101.57±814.89	10.42±0.15	1.12±0.06
Kephyes ovata	12.70	7.83	1.59	4.03	1.15	0.70	24002.70	8754.46	10.40	1.12
Lensia conoidea	9.10±0.61	5.73±0.13	1.64±0.08	4.25±0.19	1.38±0.09	0.31±0.02	9023.12±1749.58	10222.28±2034.21	9.81±0.16	1.15±0.06
Lilyopsis fluoracantha	9.86±0.77	6.77±0.54	1.89±0.24	3.97±0.26	1.19±0.08	0.19±0.01	594072.66±96123.86	250178.89±38244.55	13.62±0.16	0.52±0.03
Lilyopsis medusa	9.42	6.31	1.73	4.06	1.58	0.10	7841.90	23345.58	10.35	1.12
Lychnagalma utricularia	4.31±0.34	5.92±0.47			1.41±0.08	0.00±0.00	8642061.17±1422405.09	4940439.15±1209632.19	16.39±0.14	0.57±0.06
Marrus claudanielis	3.90±0.19	6.57±0.29	1.56±0.08	3.68±0.47	1.60±0.25	0.01±0.00	6690249.26±1488301.16	1993627.90±1037304.20	15.87±0.24	0.72±0.07
Marrus orthocanna	4.09	7.83	1.70	3.14	1.22	0.07	140678.33	45324.43	12.13	1.17
Nanomia sp	2.14±0.11	5.17±0.35	2.04±0.11	3.81±0.18	1.43±0.05	0.02±0.00	627963.84±254457.98	380184.45±168164.31	12.94±0.45	0.87±0.07
Nectadamas richardi	5.05	8.27					49421.16		9.02	0.52
Physalia physalis		1.13±0.04						231908.96±99649.17	12.02±0.41	0.28±0.02
Physonect sp	6.51	8.17	1.40	2.55	1.57	0.16	1344002.91	283820.19	14.30	0.51
Physophora gilmeri	3.88	5.93			1.46	0.02	3841887.52	1167163.94	15.43	0.52
Physophora hydrostatica	4.29±0.22	5.76±0.32	1.36±0.12	4.55±0.20	1.36±0.04	0.03±0.00	760746.88±315977.47	658017.60±226836.91	13.68±0.57	0.71±0.09
Praya dubia	10.66±1.38	8.67±0.92	1.53±0.12	4.68±0.36	1.44±0.17	0.20±0.02	508561.11±118405.79	210656.48±36776.48	13.39±0.23	0.59±0.02
Praya reticulata	13.86	8.98	1.42	4.38	1.24	0.11	1763474.33	1069367.68	14.86	0.43
Resomia dunni	6.19	8.17	2.03	2.70	1.19	0.02		7248532.33	15.80	0.40
Resomia ornicephala	4.03±0.24	6.86±0.26	1.20±0.04	3.87±0.20	1.37±0.09	0.01±0.00	1992106.63±424360.10	1571487.06±195174.19	15.02±0.16	0.51±0.03
Resomia persica	4.60	7.03	1.89	6.77	1.27	0.02		3997350.68	15.20	0.38
Rhizophysa eysenhardtii		1.20±0.08						134882.90±36730.34	11.67±0.27	0.41±0.03
Rhizophysa filiformis		1.09±0.03						47446.55±9349.78	10.67±0.23	0.26±0.02
Rosacea cymbiformis	8.09±0.32	6.08±0.70	1.51±0.20	4.00±0.67	1.27±0.10	0.22±0.03	43725.67±22556.88	43145.89±21739.40	11.10±0.53	0.84±0.14
Rosacea plicata	8.61	7.38	1.84	3.88	1.30	0.34	121646.77	20424.72	11.86	1.04
Sphaeronectes koellikeri	5.02±0.38	6.01±0.35	1.89±0.21	4.07±0.51	1.57±0.12	0.44±0.04	1525.07±119.41	703.81±35.05	7.70±0.06	2.39±0.11
Stephanomia amphytridis	3.74±0.15	7.19±0.62	1.64±0.09	3.58±0.26	1.29±0.05	0.01±0.00	65597086.06±7019318.87	27409899.85±3280806.25	18.06±0.29	0.25±0.01
Stephanophyes superba	11.45±1.26	6.28±0.58	1.44±0.23	5.08±0.54	1.36±0.24	0.10±0.02	137995.93±56959.00	146447.73±10020.17	12.54±0.17	0.69±0.04
Sulculeolaria quadrivalvis	6.69±0.37	5.34±0.42	1.77±0.21	3.86±0.32	1.37±0.07	0.27±0.03	3082.84±735.80	4551.03±1254.02	8.85±0.21	1.42±0.14
Thermopalia taraxaca	2.41		2.38	4.37	1.45				8.15	
Vogtia glabra	4.67	5.23	2.08	4.82	1.58	0.19	298333.17	71426.76	12.82	0.72
Vogtia serrata	6.96±0.28	7.72±0.60	1.69±0.08	3.88±0.31	1.50±0.05	0.13±0.02	621637.11±99451.35	264272.03±43860.12	13.65±0.15	0.67±0.02
Vogtia spinosa	5.28	6.98	1.77	3.53	1.54	0.14	337630.32	119686.37	13.03	0.82
